# Therapeutic Potential of Lentiviral miR‐200a Mimics in Regulating Fibrinolysis and EMT Markers During Pulmonary Fibrosis

**DOI:** 10.1096/fba.2025-00276

**Published:** 2026-04-17

**Authors:** T. M. Jeena, C. Rakshitha, Akarsha B. Jain, Vaishnavi, Fathima Razana, Aleena Varughese, M. Fathimath Muneesa, Yashodhar P. Bhandary

**Affiliations:** ^1^ Cell Biology & Molecular Genetics Division, Yenepoya Research Centre, Yenepoya Deemed to be University Mangalore Karnataka India; ^2^ The University of Texas Health Science Centre Fort Worth Texas USA; ^3^ Department of Bioscience Mangalore University Mangalore Karnataka India; ^4^ IQRAA Hospital & Research Centre Calicut Kerala India; ^5^ Specialized Research Unit, Yenepoya Medical College & Hospital, Yenepoya Deemed to be University Mangalore Karnataka India

**Keywords:** ECM, fibrosis regulation, IPF, lentiviral transfection, miR‐200a, MMPs

## Abstract

Idiopathic pulmonary fibrosis (IPF) is a chronic lung disease characterized by the excessive accumulation of collagen‐rich extracellular matrix (ECM), leading to the replacement of normal lung architecture. This pathological remodeling is primarily caused by the epithelial‐to‐mesenchymal transition (EMT), in which epithelial cells lose their polarity and adhesion and acquire mesenchymal characteristics that encourage the deposition of ECM and the growth of fibrotic tissue. The disruption of ECM homeostasis caused by dysregulated MMP‐2 and MMP‐9 activity in IPF paradoxically promotes abnormal tissue remodeling and fibrosis. IPF's unknown etiology, delayed diagnosis, and lack of effective treatments point to a crucial knowledge gap regarding ECM‐ and EMT‐driven fibrosis. The microRNA‐200 (miR‐200) family has been found to be important EMT regulators in recent research, suggesting that they may be able to influence the course of fibrosis. Hereby the current study provides an insight on the role of miR‐200a in regulating MMP‐2 and MMP‐9 in bleomycin (BLM)‐induced lung fibrosis using both in vitro (A549 cells) and in vivo (C57BL/6 mice) models. A549 cells were transfected with a synthetic miR‐200a mimic, and MMP expression was analyzed using RT‐qPCR. In vivo, mice were intranasally administered a lentiviral vector expressing miR‐200a prior to BLM induction, followed by tissue analysis at days 14 and 21 using histological stains and immunofluorescence. Gene and protein expression were quantified via RT‐qPCR and western blotting. Our findings indicate that miR‐200a mitigates fibrosis by downregulating MMPs and PAI‐1 while upregulating uPA and uPAR, suggesting a protective role of miR‐200a and its potential as a therapeutic target for pulmonary fibrosis.

## Graphical abstract

The graphical abstract contrasts normal and fibrotic lungs, illustrating how miR‐200a mimics suppress PAI‐1 and EMT markers, restore fibrinolysis, and reduce MMP‐driven ECM deposition. This schematic highlights the therapeutic potential of miR‐200a in mitigating pulmonary fibrosis progression.

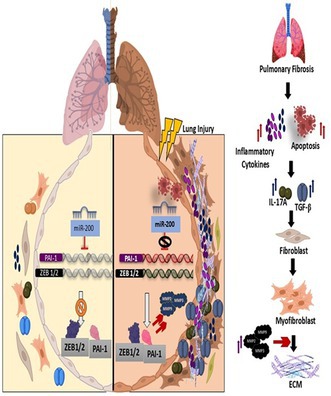

## 1 Introduction

Idiopathic pulmonary fibrosis (IPF) is a broad term for a family of diseases where normal lung tissue architecture becomes replaced with scar tissue. IPF is characterized by the developmental process defined by the loss of epithelial characteristics and acquisition of mesenchymal phenotype called epithelial‐mesenchymal transition (EMT) [1, 2, 3]. They are generally marked by collagen deposition and fibroblast proliferation producing myofibroblasts in lung tissue responsible for alveolar fibrotic changes leading to decreased gas exchange and pulmonary distress [4, 5]. IPF is a complicated and frequently lethal lung disease that causes lung tissue to gradually scar, which significantly impairs lung function. The pathophysiology of IPF is still mostly unknown, which makes diagnosis and successful therapy more difficult. In Koreans aged ≥ 40, the incidence of IPF rose from 2.5 to 3.8 cases per 100,000 between 2011 and 2016. Between 2008 and 2019, the mortality rate for men over 85 increased by 6.5% annually. IPF‐related mortality increased by 72% between March and April 2020, suggesting that this cohort is more vulnerable. In northeastern Italy, elderly men's IPF mortality was rising. Since less than two out of every three IPF‐related deaths were directly attributable to this condition, the impact of IPF was greater than routine statistics would have indicated [6, 7, 8].

The pathophysiology of IPF is facilitated by impaired fibrinolytic activity in the lungs, which permits chronic fibrin accumulation. This leads to fibroblast activation, excessive collagen deposition, and progressive lung tissue scarring. The fibrinolytic system includes plasminogen, an inactive proenzyme that transforms into plasmin, an active enzyme that breaks down fibrin. This system serves as a safeguard during fibrin deposition. Three key molecules constitute the fibrinolytic system: Urokinase Plasminogen Activator (uPA), urokinase plasminogen activator receptor (uPAR), and Plasminogen Activator Inhibitor‐1 (PAI‐1). uPA and uPAR primarily facilitate the conversion of plasminogen to plasmin, leading to the breakdown of the extracellular matrix (ECM). Reduced plasmin production and poor fibrin clearance result from PAI‐1's modulation of fibrinolytic activity through binding to and suppressing uPA and uPAR [9, 10, 11].

Normal organ function depends on the ECM, a tissue‐specific macromolecular framework that gives tissues physical support. IPF is primarily caused by ECM deposition, which results in aberrant lung architecture and functioning. The main process is caused by oxidative stress, inflammation, persistent epithelium damage, and altered signaling, particularly transforming growth factor‐beta (TGF‐β), which causes overactive fibroblasts and myofibroblasts to produce excess collagens (types I, III, and VI) [12]. By enhancing tissue remodeling and fibrosis through bioactive peptides and altered growth factor interactions, matrix anomalies serve as both a driver and a regulator of cellular responses, ultimately sustaining the progression of the disease. Myofibroblast formation and ECM deposition in IPF are facilitated by the process of epithelial cells losing their features and acquiring mesenchymal qualities, or EMT [13, 14]. The molecular processes of EMT include abnormal activation of signaling pathways, including Wnt/β‐catenin and TGF‐β, which use paracrine signaling to promote fibrosis and fibroblast activation [15, 16]. EMT promotes fibrosis and tissue remodeling in IPF by interfering with normal lung healing. This process produces a profibrotic milieu and is impacted by senescence, mechanical stress, and damage to epithelial cells [17]. New diagnostic indicators and treatments that target fibrosis in IPF may result from a better understanding of EMT's function. An uncontrolled wound‐healing process and a disrupted fibrinolytic system result in excessive ECM and EMT deposits, which are significant in conditions like PF [18, 19]. Researchers aim to understand the molecular mechanisms behind fibrosis progression to maintain the fibrinolytic system's homeostasis.

Recent studies highlight the role of microRNAs (miRNAs) in fibrosis development. miRNAs function by binding to the complementary regions in target mRNAs' 3′UTR to control gene expression. Through the degradation of the mRNA or the inhibition of its translation, this binding results in the repression of protein synthesis [20]. These conserved noncoding small molecules regulate oncogenic processes, tumor suppression, alveolar EMT, and epithelial cell morphology. Additionally, the miR‐200 family was the first oncogenic miRNAs (OncoMiR) to be discovered and promotes TGF‐β‐induced EMT [21, 22, 23]. It has been revealed that the miR‐200 family members (miR‐200a/200b/200c/141/429) reduce invasion, cell migration, and EMT by targeting Zinc Finger E‐box Binding Homeobox‐1 (ZEB1) and ZEB mRNA through two E‐cadherin repressor levels [24, 25, 26]. There have also been reports of miR‐200 family inhibition in situations when the DNA locus is hypermethylated [27, 28]. On the other hand, miR‐200c has been shown to reduce metastasis in A549 NSCLC cell lines [29, 30, 31].

The zinc‐dependent endopeptidases known as matrix metalloproteinases (MMPs) break down ECM components and are essential for wound healing, tissue remodeling, and other pathological processes. Through their modulation of the lung microenvironment, MMPs contribute to the advancement of PF. Excessive ECM deposition brought on by dysregulated MMP activity can cause fibrosis and compromised lung function. Research has demonstrated that some MMPs, including MMP‐2 and MMP‐9, are elevated in IPF and are linked to the prognosis and severity of the illness [32]. Furthermore, the pathophysiology of PF is significantly influenced by the interaction between MMPs and miRNAs. MMP expression and activity are influenced by the differential expression of miRNAs in IPF lung tissue, which in turn affects fibrotic processes [33]. For example, by controlling MMPs and other fibrosis‐related genes, certain miRNAs can either stimulate or prevent fibrosis.

Bleomycin (BLM) is an antitumor antibiotic used in the treatment of cancers such as lymphomas, but its pulmonary toxicity limits clinical use, with fibrosis occurring in a subset of patients due to insufficient enzymatic inactivation in the lungs. By generating reactive oxygen species, BLM induces DNA damage, epithelial injury, and fibrotic remodeling, making it a well‐established experimental model for idiopathic pulmonary fibrosis [34, 35]. This model reproduces key pathological features of IPF, including inflammation and collagen deposition, and has been instrumental in the preclinical evaluation of antifibrotic therapies such as pirfenidone and nintedanib. Building on this framework, the present study investigates the role of miR‐200a in regulating MMP‐2 and MMP‐9 in bleomycin‐induced lung fibrosis using both in vitro and in vivo approaches.

Our study shows that miR‐200a plays a protective role in pulmonary fibrosis by suppressing MMP‐2, MMP‐9, and PAI‐1 while enhancing uPA and uPAR expression. Using both in vitro and in vivo models, our findings indicate that miR‐200a helps restore fibrinolytic balance, reduces excessive ECM deposition, and mitigates fibrotic progression. These results suggest that miR‐200a may serve as a promising therapeutic target for idiopathic pulmonary fibrosis and provide new insight into the molecular mechanisms underlying EMT‐ and ECM‐driven fibrosis.

## 2 Materials

### 2.1 Media

Dulbecco's Modified Eagle Medium (Himedia, India), Fetal bovine serum (Himedia, India), Antibiotic solution (Himedia, India).

### 2.2 Drugs

Bleomycin (BIOCHEM pharmaceutical industries limited, India), shMIMIC Mouse Inducible microRNA mmu‐miR‐200a‐3p mCMV‐TurboGFP (Cat No: VSM6920‐22465604429349990), SMARTvector Inducible Non‐targeting Control (Vector control) (Cat No: VSM6390) (Figure S1), Ketamine.

### 2.3 Chemicals

Sodium chloride (Molecular grade, Himedia, India), Tris‐buffer (Molecular gradeHimedia, India), Tris‐Hcl (Molecular grade, Himedia, India), Sodium citrate (Molecular grade, Himedia, India). Sodium do‐decyl sulphate (Molecular grade, Himedia, India), Glycine (Molecular grade, Himedia, India), Phosphate buffered saline (Molecular grade, Himedia, India), Tween‐20 (Molecular grade, Himedia, India), and Commasiebriliant blue G‐250 (Molecular grade, Himedia, India).

### 2.4 Antibodies

Anti‐β‐actin antibody (Sigma‐Aldrich, Japan), Anti‐IL‐17A antibody (CST, USA), Anti‐PAI‐I antibody (Bio‐Rad, USA), Anti‐uPA antibody (R&D Systems, USA), Anti‐uPAR antibody (R&D Systems, USA), Anti‐TGF‐β1 antibody (CST, USA), MMP‐2 (R&D Systems, USA), MMP‐9 (R&D Systems, USA), ZEB1 (R&D Systems, USA), Fibronectin (CST, USA), Vimentin (CST, USA), Collagen (R&D Systems, USA), and ZEB2 (R&D Systems, USA) (Antibodies used in this study are listed with their RRIDs in Table S1).

## 3 Methods

### 3.1 In Vitro Studies

#### 3.1.1 Cell Line and Culture Conditions

Human alveolar basal epithelial cells (A549, RRID:CVCL_0023), acquired from the National Centre for Cell Sciences (NCCS), Pune, were cultured in Dulbecco's Modified Eagle's Medium (DMEM) supplemented with 10% fetal bovine serum and 1% antibiotic‐antimycotic solution. The cells were incubated at 37°C in a humidified atmosphere containing 5% CO_2_. The cell culture was regularly tested for mycoplasma contamination and stress‐related morphological changes. Only healthy cells were used for experiments after completing five consecutive passages.

#### 3.1.2 Experimental Fibrosis Model for miR‐200a

To establish the experimental fibrosis model and evaluate the effect of miR‐200a, the A549 cells were allocated into six groups: a vehicle control treated with 25 ng/mL, a negative control mimic at 25 ng/mL diluted in nuclease‐free water, miR‐200a mimic alone at 25 ng/mL, a combination of negative control mimic and bleomycin (BLM) at 25 ng/mL and 40 μg/mL respectively, miR‐200a mimic with BLM at 25 ng/mL and 40 μg/mL respectively, and a BLM‐only group receiving 40 μg/mL. After 24 h of BLM exposure, cells were transfected with the designated mimics. Seventy‐two hours after BLM treatment, the cells were harvested, and expression levels of matrix metalloproteinases (MMPs) were analyzed using RT‐PCR.

#### 3.1.3 Gene Expression Studies

RNA was isolated from lysed cells using Tri‐reagent (Sigma‐Aldrich), followed by complementary DNA synthesis using the iScript cDNA Synthesis Kit (BIO‐RAD), adhering to the supplier's instructions. Quantitative PCR was carried out using TB Green PCR Master Mix (Takara Bio USA). All the primers are custom designed and the primer sequences are listed in Table 1.

**TABLE 1:** List of human primers used for in vitro study.

Gene	Species	Primer pairs (5′‐3)
MMP‐2	Human	F: TATGGCTTCTGCCCTGAGAC
		R: CACACCACATCTTTCCGTCA
MMP‐9	Human	F: GAAGCTCGGCTGGAGAGAAG
		R: TTTGAGTCCGGTGGACGATGC
β‐Actin	Human	F: TTAATTTCTGAATGGCCCAG
		R: GACCAAAGCCTTCATACATC

### 3.2 In Vivo Studies

#### 3.2.1 Animals

Male C57BL/6 mice (weighing 25 ± 5 g and aged 7–8 weeks) were acquired from Adita Biosys Private Limited in Tumakuru, Karnataka, India. The mice were given free access to food and water. The animals were housed in a sterile polypropylene cage, and the bedding was made of sterile rice husk. The experiments were carried out after receiving approval from the Institutional Animal Ethics Committee (IAEC), Institutional Biosafety Committee (IBSC), Yenepoya (Deemed to be University), and the CPCSEA (Committee for Control and Supervision of Experiments on Animals) guidelines for animal experimentation. The animals were handled and cared for in accordance with these guidelines.

Inclusion criteria:

- Healthy male C57BL/6 mice aged 7–8 weeks and weighing 25 ± 5 g.
- Mice with no signs of illness or injury prior to the study.

Exclusion criteria:

- Mice exhibiting signs of illness, injury, or abnormal behavior during the acclimatization period.
- Mice with any pre‐existing respiratory conditions or weight outside the specified range.

Attrition statement:

The general attrition throughout the study was less than 10%. The minimal loss of animals was caused by inter‐male aggression, and this was due to occasional fighting‐related mortality. No animals were excluded based on treatment‐related adverse effects.

### 3.3 In Vivo Models for PF

Following a 14 day isolation period, mice were randomly allocated to experimental groups after acclimatization to reduce selection bias. Six animals per group were used, consistent with previous fibrosis studies that demonstrated adequate sensitivity to detect biologically relevant differences. A formal power calculation was not performed for this study. The mice were divided into six groups (Table 2): Control (untreated), Vector control (SMART vector inducible non‐targeting control) (Table S2), BLM (Bleomycin‐only), BLM + Vector control, LV‐miR‐200a, and BLM + LV‐miR‐200a. On the 0th day of treatment (day 18 of the study), BLM (3 units/kg) was administered intranasally to the BLM, BLM + Vector control, and BLM + LV‐miR‐200a groups. Additionally, on the same day, 2 × 10^6^ IFUs of the SMART vector were administered intranasally to the Vector control group, and 2 × 10^6^ IFUs of LV‐miR‐200a were administered to the LV‐miR‐200a group.

**TABLE 2:** In vivo experimental grouping and treatment dosages.

Group	Treatment details	BLM dose	Vector dose	Sacrifice days
Control	No treatment	None	None	Day 14 and Day 21
Vector control	SMART vector only	None	2 × 10^6^ IFUs (Day 0 and Day 1)	Day 14 and Day 21
BLM‐only	Bleomycin only	3 U/kg	None	Day 14 and Day 21
BLM + Vector control	Bleomycin + SMART vector	3 U/kg	2 × 10^6^ IFUs (Day 0 and Day 1)	Day 14 and Day 21
LV‐miR‐200a	Lentiviral miR‐200a only	None	2 × 10^6^ IFUs (Day 0 and Day 1)	Day 14 and Day 21
BLM + LV‐miR‐200a	Bleomycin + Lentiviral miR‐200a	3 U/kg	2 × 10^6^ IFUs (Day 0 and Day 1)	Day 14 and Day 21

After 24 h (day 1 of treatment), further doses were given. The Vector control group and the BLM + Vector control group each received 2 × 10^6^ IFUs of the SMART vector. Similarly, the LV‐miR‐200a group and the BLM + LV‐miR‐200a group were administered an additional 2 × 10^6^ IFUs of LV‐miR‐200a (Figures S2 and S3). The Control group received no treatment throughout the study. Mice were then sacrificed on the 14th and 21st days post‐treatment for sample collection and analysis. Researchers performing sample analysis and histological scoring were blinded to group allocation to reduce observational bias.

### 3.4 Isolation of Protein and Quantification

Mice lungs were collected and subjected to tissue homogenization using an extraction buffer (Sodium EDTA pH 7.9). Lung homogenates were centrifuged at 10000 g for 15 min at 4°C. The supernatant was preserved at −80°C. The protein concentration in lung homogenate samples was estimated using Bradford's method.

SDS‐Polyacrylamide gel electrophoresis was performed with the protein isolates from different treatment groups using 10% gels. The proteins were electroblotted on a polyvinylidene difluoride (PVDF) membrane and blocked with 2% BSA in tris‐buffered saline with tween‐20 (TBST). The PVDF membrane was incubated with primary antibodies with a dilution of 1:1000 at 4°C overnight. This is followed by incubation with goat anti‐rabbit horseradish peroxidase (HRP) conjugated secondary antibody with a dilution of 1:5000 for 1 h at room temperature. Protein bands were visualized using enhanced chemiluminescence; further, the blots were visualized in Vilbert chemiluminescence (Pierce ECL western blotting substrate, Thermo Fisher Scientific, USA).

### 3.5 Histopathology and Immunostaining

After inflating the mice's lungs with 4% formalin, they were incubated for 96 h. The lung tissues that had been fixed in formalin underwent tissue processing before being embedded in melted paraffin wax. Sliced into 3.5 μm slices, formalin‐fixed paraffin‐embedded (FFPE) tissues were submerged in one to three separate xylene immersions to extract paraffin.

To detect histological alterations, the lung tissue slices were rehydrated using various alcohol grades and stained with hematoxylin and eosin (H&E). Sections of FFPE lung tissues measuring 3.5 μm were cut and treated appropriately. In order to visualize the targeted protein expression, which was standardized with DAPI, these sections were then subjected to immunofluorescent (IF) labelling using a secondary antibody coupled to Alexa‐488.

Additionally, Masson Trichrome staining was done on the lung sections to determine the collagen content. Collagen fibers, which stained blue, were distinguished from other tissue constituents with the aid of this approach. This made the fibrotic alterations in the lung tissues clearly visible.

### 3.6 Gene Expression Studies

Tri‐reagent (Sigma‐Aldrich) was used to extract total RNA from lung tissues in accordance with the manufacturer's instructions, and the iScript cDNA Synthesis Kit (BIO‐RAD) was used to reverse‐transcribe the extracted RNA. Real‐time PCR was performed using the Takara TB Green PCR Master mix. All the primers are custom designed and the primer sequences are listed in Table 3.

**TABLE 3:** Gene‐specific primers for in vivo studies used for RT‐PCR.

Gene	Species	Primer pairs (5′‐3)
uPA	Mouse	F: AGAGTCTGAAAGTGACTATCTC
		R: CCTTCGATGTTACAGATAAGC
Vimentin	Mouse	F: GGACCAGCTAACCAACGACA
		R: AAGGTCAAGACGTGCCAGAG
PAI‐1	Mouse	F: AGCAACAAGTTCAACTACAC
		R: CTTCCATTGTCTGATGAGTTC
MMP‐2	Mouse	F: TATGGGAACGCTGATGGCGA
		R: AACAAGGCTTCATGGGGGCA
MMP‐9	Mouse	F: CTTCTGGCGTGTGAGTTTCCA
		R: ACTGCACGGTTGAAGCAAAGA
Fibronectin	Mouse	F: CCAACTCCTTGCTGGTGTCA
		R: TTGGGGAAGCTCATCTGTCT
Collagen	Mouse	F: GCCAAGACGAAGACATCCCA
		R: GGCAGTTCTTGGTCTCGTCA
β‐Actin	Mouse	F: TTAATTTCTGAATGGCCCAG
		R: GACCAAAGCCTTCATACATC

### 3.7 Real‐Time PCR for miR‐200a

Total RNA, including small RNAs, was isolated from lung tissue using TRIzol reagent (Thermo Fisher Scientific) following the manufacturer's protocol. RNA concentration and purity were determined spectrophotometrically, and integrity was confirmed prior to downstream analysis. Complementary DNA (cDNA) was synthesized using the TaqMan MicroRNA Reverse Transcription Kit (Thermo Fisher Scientific) with stem‐loop primers specific for hsa‐miR‐200a (Assay ID: 002300) and U6 snRNA (Assay ID: 001973) as the endogenous control. Reverse transcription reactions were prepared in a 15 μL volume and subjected to the following thermal cycling program: 30 min at 16°C, 30 min at 42°C, and 5 min at 85°C. Quantitative PCR was performed on an Applied Biosystems real‐time PCR instrument using the TaqMan Fast Universal PCR Master Mix (2X), AmpErase UNG (Thermo Fisher Scientific). Each 20 μL reaction contained PCR Master Mix, the respective TaqMan MicroRNA Assay probe, and cDNA template. The cycling conditions were as follows: 50°C for 2 min (UNG activation), 95°C for 10 min (enzyme activation), followed by 40 cycles of 95°C for 15 s and 60°C for 60 s. Relative expression levels of miR‐200a were calculated using the comparative Ct (^ΔΔ^Ct) method, with normalization to U6 snRNA.

### 3.8 Statistical Analysis

All data are expressed as mean ± SD. Comparisons between animal groups were performed using one‐way ANOVA or Student's *t*‐test, depending on the experimental design. Prior to analysis, assumptions of normality (Shapiro–Wilk test) and equality of variances (Levene's test) were verified. Post hoc comparisons following ANOVA were conducted using Tukey's test. Statistical analyses were carried out using SPSS software, version 22 (RRID:SCR_002865). A *p* value < 0.05 was considered statistically significant.

## 4 Results

### 4.1 miR‐200a Alleviates Bleomycin‐Induced MMP‐2 and MMP‐9 Expression in A549 Cells

**FIGURE 1:**
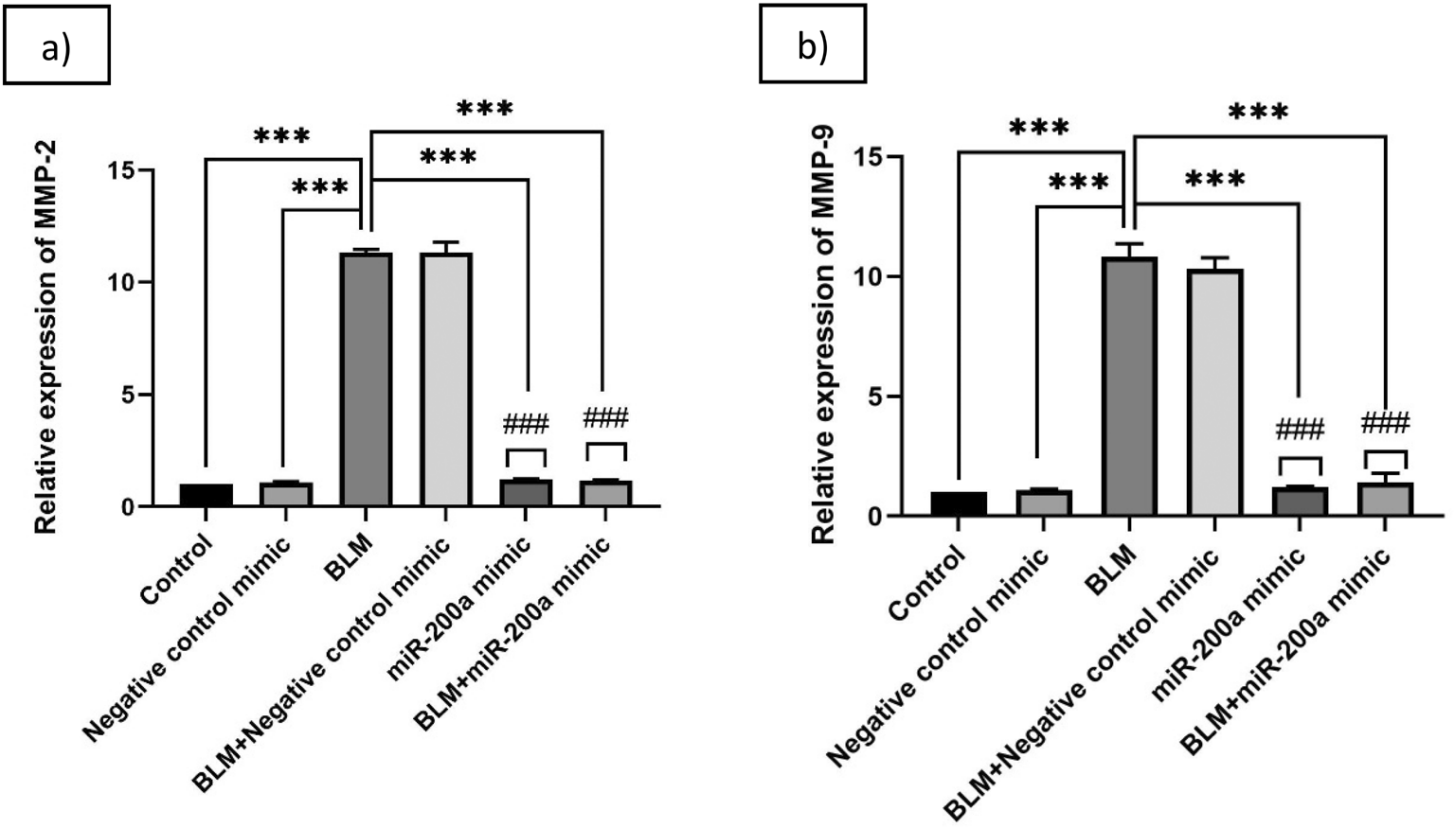
Total RNA was isolated from the A549 cells and subjected to RT‐PCR. (a) mRNA levels of MMP‐2 and (b) MMP‐9. Relative gene expressions were compared with β‐Actin, and the relative fold changes were represented graphically. Level of significance: *p < 0.001 compared to BLM; #*p* < 0.001 compared to BLM + Negative control mimic exposure (Mean ± SD, *n* = 6).

Fibrosis, which is marked by excessive ECM deposition that results in tissue scarring and organ failure [36, 37]. It has been reported that the miR‐200 family, which consists of miR‐200a, miR‐200b, miR‐200c, miR‐141, and miR‐429, is involved in a number of different aspects, such as tumor angiogenesis, tumor chemotherapy resistance, and EMT [38, 39, 40]; these characteristics indicate to be primarily caused by the regulation of expression of some significant target genes by miR‐200 family members [38]. Although several members of the miR‐200 family are known to influence EMT and fibrosis, we focused on miR‐200a because prior studies, including our own work, have shown that it consistently regulates key mediators of fibrotic remodeling. Both miR‐200a and miR‐200b were previously demonstrated to reduce acute lung injury through modulation of the fibrinolytic system and EMT pathway, but miR‐200a displayed a stronger and more reproducible effect on MMP‐2 and MMP‐9 expression. These findings provided a clear rationale for selecting miR‐200a for further investigation in the BLM‐induced fibrosis model. To investigate the modulatory effect of miR‐200a on fibrotic responses, A549 cells were treated with BLM (40 μg/mL) alone, in combination with miR‐200a mimics (25 ng/mL) and a negative control mimic (25 ng/mL). Quantitative real‐time PCR analysis revealed that BLM exposure significantly elevated the mRNA expression levels of MMP‐2 with a fold change of ~11.3 (*p* < 0.001) (Figure 1a) and ~10.8 (*p* < 0.001) of MMP‐9 (Figure 1b) in comparison with the untreated condition. The overexpression of miR‐200a in BLM‐induced IPF reduced the MMP‐2 and MMP‐9 mRNA expression with a fold change of ~1.2 and ~1.1 (*p* < 0.001) respectively. This downregulation of MMPs in miR‐200a with BLM indicates that miR‐200a may exert a suppressive role on BLM‐induced ECM remodeling by targeting MMP‐2 and MMP‐9 transcripts in in vitro systems.

### 4.2 LV‐miR‐200a Mimic Restores Levels of miR‐200a in BLM‐Induced IPF

**FIGURE 2:**
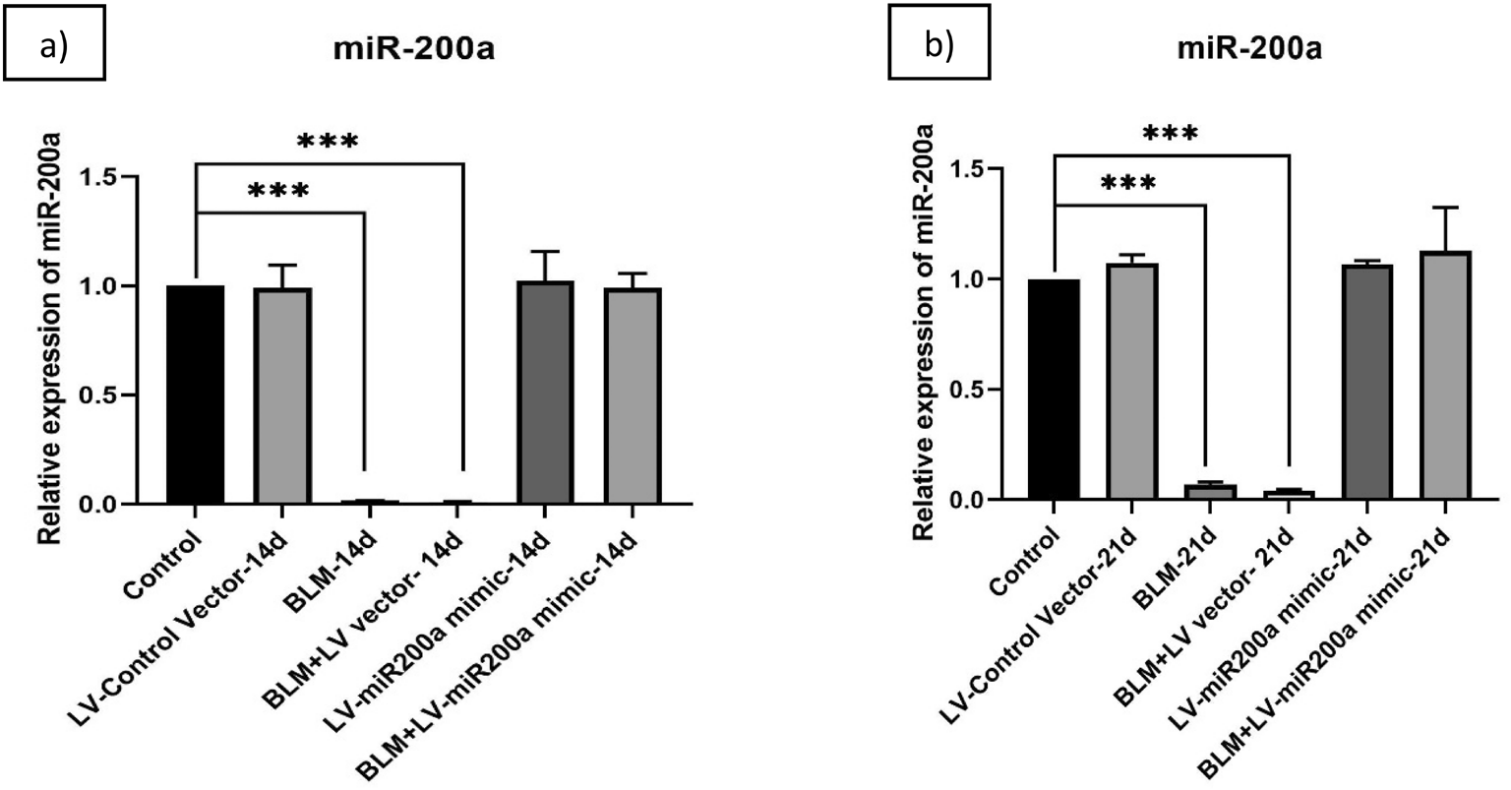
C57BL/6 mice were exposed to BLM (3 units/kg body wt), and then treated with LV‐miR‐200a after 24 h. Mice were sacrificed after 14 and 21 days of BLM exposure and lungs of the mice were collected. Total RNA was isolated from the lung homogenate and analyzed by RT‐PCR for (a) miR‐200a for 14th day (b) miR‐200a for 21st day. Relative gene expressions were compared with U6 snRNA, the relative fold changes are represented graphically. Level of significance: *p < 0.001 compared to BLM exposure (Mean ± SD, *n* = 6).

In renal fibrosis, miR‐200a is said to have a significant impact on the development and progression of TGF‐β‐dependent EMT and fibrogenesis. The miR‐200a expression is notably downregulated, and it was induced through a lentiviral vector, enabling the suppression of TGF‐β‐dependent EMT [41]. To explore its therapeutic potential in IPF, we used lentiviral vector to restore the miR‐200a expression in a BLM‐induced IPF mouse model. The C57BL/6 mice were exposed to BLM at a dose of 3 units per kilogram of body weight and treated with LV‐miR‐200a mimic 24 h after BLM exposure. To confirm successful transfection of miR‐200a via LV vectors, the lung tissues were harvested on days 14 and 21 post BLM exposure, and miR‐200a expression was quantified using RT‐qPCR. Results showed that miR‐200a expression remained significantly reduced in BLM‐administered mice with ~0.02 and ~0.07 (*p* < 0.001) fold change on the 14th (Figure 2a) and 21st (Figure 2b) days respectively. Lentiviral delivery of miR‐200a mimics effectively restored miR‐200a levels in lung tissue, demonstrating successful incorporation of this therapeutic approach. These findings indicate that miR‐200a supplementation can counteract BLM‐induced downregulation and modulate pathways associated with fibrosis, consistent with previous observations in ALI models.

### 4.3 miR‐200a Overexpression Ameliorates Lung Injury in BLM‐Induced Pulmonary Fibrosis

**FIGURE 3:**
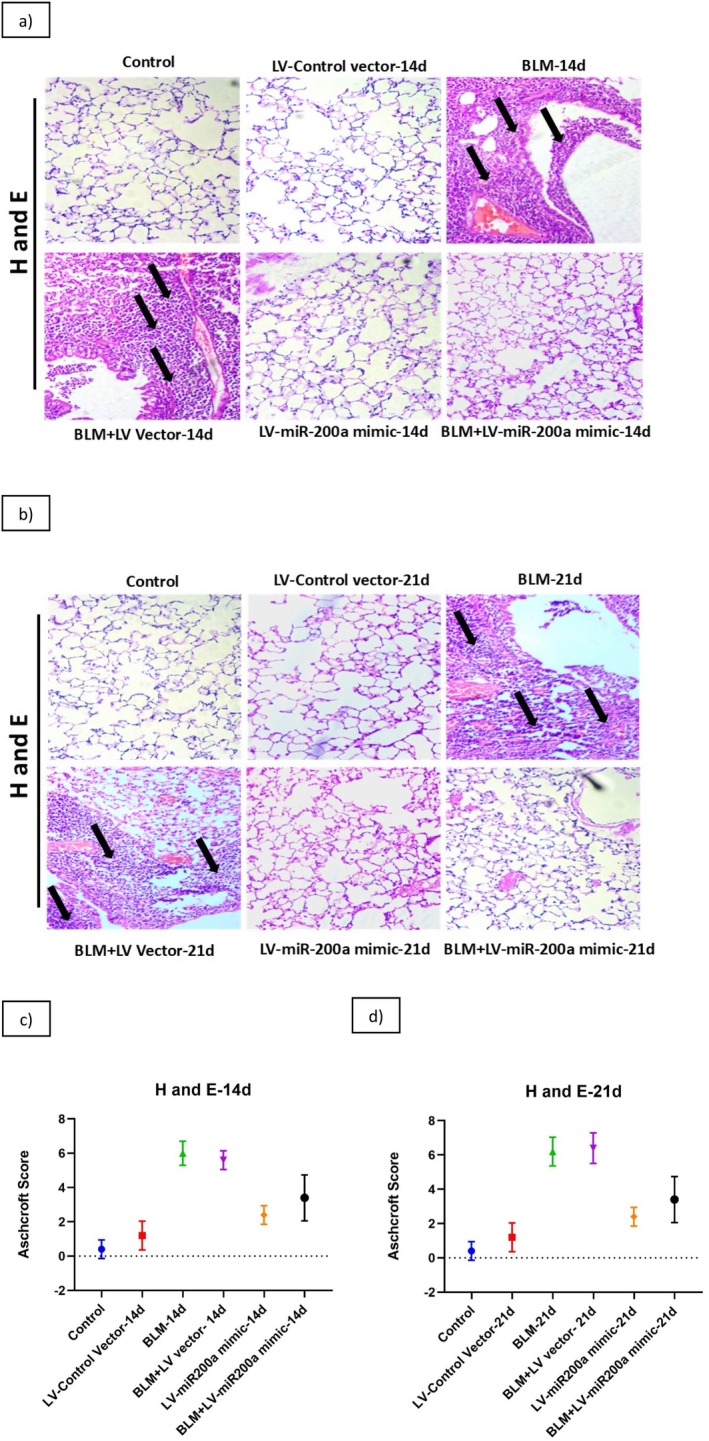
C57BL/6 mice were exposed to BLM (3 units/kg body wt), and then treated with LV‐miR‐200a mimics after 24 h post exposure. Mice were sacrificed after 14th day and 21st day of BLM exposure and lungs of the mice were collected. Mice lung tissue sections (3.5 μm) were subjected for H&E staining which helps to visualize cellular structures and fibrosis in IPF lung tissue for diagnosis and assessment. (a) lung morphology on day 14 post BLM, (b) lung morphology on day 21 post BLM, (c) Ashcroft scoring on day 14 (d) Ashcroft scoring on day 14; (LV stands for Lentiviral Vector). Magnification ×400.

In our previous studies, we found that miR‐200a and miR‐200b regulate ALI by recovering the fibrinolytic system. We also know that LV‐miR‐200a mimics reduce myofibroblast activation and decrease ECM production by restoring miR‐200a levels, which serves as a therapeutic strategy for fibrosis [42]. We were interested in investigating the role of miR‐200a in IPF, and our current study explores the therapeutic potential of miR‐200a mimics in a BLM‐induced model of IPF in C57BL/6 mice. BLM administration initiated fibrosis by inducing lung tissue damage, and the morphology has been observed by H&E staining. The normal alveolar morphology was significantly disrupted in BLM‐treated mice, evident by thickened interalveolar septa, collapsing alveolar gaps, and inflammatory cell infiltration (Figure 3). After administering LV‐miR‐200a mimics 24 h after BLM treatment, we observed a marked improvement in histological features on day 14 (Figure 3a) and 21 (Figure 3b), characterized by the lung architecture appearing comparable with the control group. Semiquantitative assessment using Ashcroft scoring confirmed that bleomycin increased the severity of fibrosis at day 14 (Figure 3c) and 21 (Figure 3d), while miR‐200a mimic treatment reduced fibrosis scores and improved tissue structure. These findings demonstrate that miR‐200a supplementation can mitigate bleomycin‐induced fibrotic remodeling and support its therapeutic potential in pulmonary fibrosis.

### 4.4 LV‐miR‐200a Mimics Reduce Deposition of Collagen and Fibrosis in Bleomycin‐Induced IPF to Restore Lung Architecture

**FIGURE 4:**
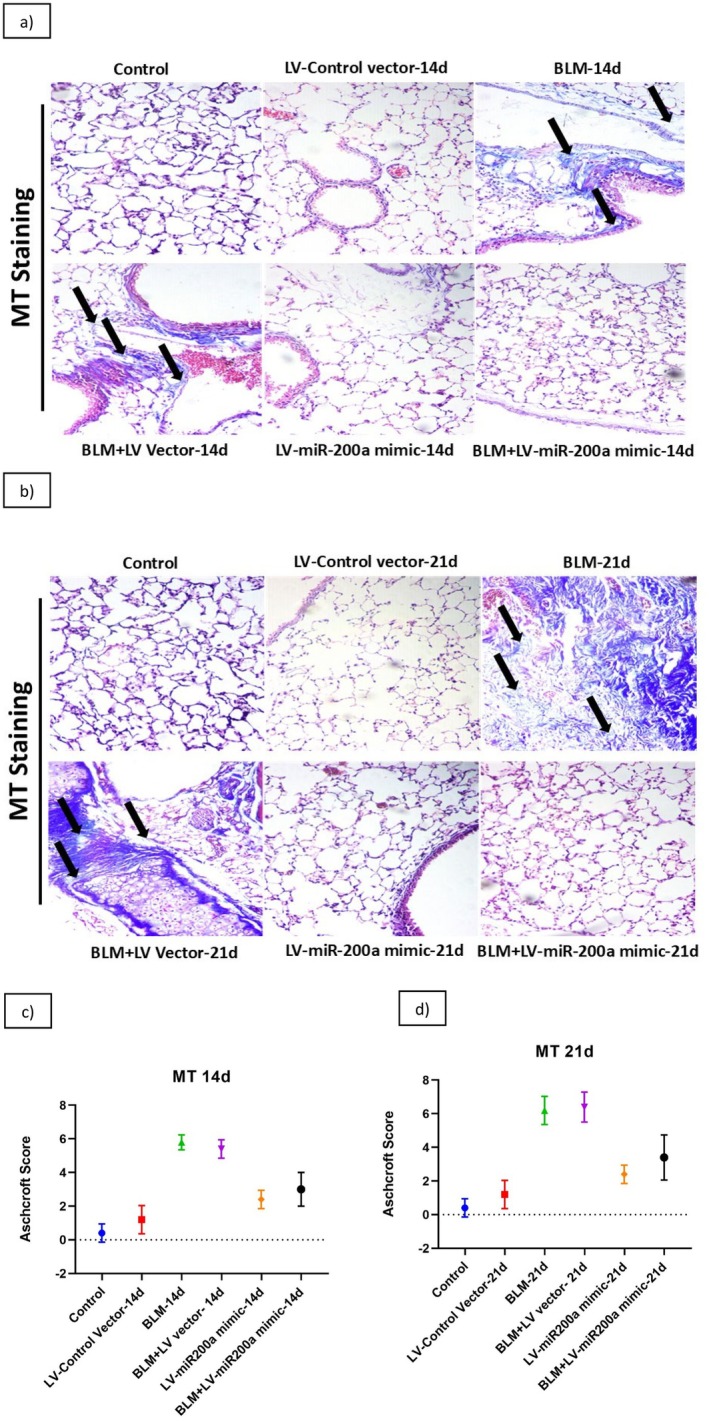
C57BL/6 mice were exposed to BLM (3 units/kg body wt), and then treated with LV‐miR200a after 24 h. Mice were sacrificed after 14 and 21 days of BLM exposure and the lungs of the mice were collected. Mice lung tissue sections (3.5 μm) were subjected to Masion and Trichrome staining to highlight collagen deposition, aiding in fibrosis assessment in IPF lung tissue. (a) lung morphology on day 14 post BLM, (b) lung morphology on day 21 post BLM, (c) Ashcroft scoring on day 14 (d) Ashcroft scoring on day 14; (LV stands for Lentiviral Vector). Magnification ×400.

Lung dysfunction and scarring in IPF are caused by collagen deposition. To further evaluate the therapeutic effect of LV‐miR‐200a mimics on BLM‐induced IPF on collagen deposition, we performed Masson's Trichrome (MT) staining on lung tissues at 14‐ and 21‐day post‐treatment. MT staining clearly illustrated significant fibrosis in the lung tissues of mice treated with BLM, characterized by disrupted tissue architecture as observed using H&E staining and extensive collagen deposition. At 14 days of BLM treatment following the administration of LV‐miR‐200a mimics after 24 h, a notable reduction in collagen deposition was observed in the lung tissues (Figure 4a). By 21 days, the lung tissues of treated mice exhibited near‐normal architecture, with minimal fibrosis and improved structural integrity compared to the BLM‐treated group (Figure 4b). These findings at both time points align with the results of H&E staining, highlighting the potent antifibrotic effects of LV‐miR‐200a mimics over time. Histological evaluation using the Ashcroft scoring revealed the IPF revealed marked lung architecture damage and increased fibrosis severity in 14th (Figure 4c) and 21st day (Figure 4d) of BLM‐treated mice, and in contrast, overexpression of miR‐200a reduced the severity and recovered the lung morphology resulting in lower Ashcroft scores. By significantly reducing collagen deposition and promoting tissue repair during the treatment, LV‐miR‐200a mimics offer a promising approach to mitigating fibrosis and improving lung health.

### 4.5 miR‐200a Modulates Gene Expressions of Fibrinolytic System, ECM, and EMT in Pulmonary Fibrosis

With increased PAI‐1 and decreased uPA levels, the fibrinolytic system becomes imbalanced in IPF, thereby impairing fibrin breakdown and leading to excessive fibrin buildup. By increasing ECM accumulation and EMT, this imbalance encourages fibrosis. Reduced lung function and elevated pulmonary arterial pressure are correlated with these alterations. All things considered, the dysregulated fibrinolytic system is essential for the advancement of lung damage and fibrosis, which greatly increases the severity and development of IPF [43]. Consequently, ECM and EMT upregulation were observed, aligning with the fibrotic phenotype characteristics of IPF. The alveolar epithelial cells go through a mesenchymal transition, which leads to the buildup of fibroblasts. In the ECM, collagen and laminin restrict this transition, but fibronectin and fibrin stimulate it by activating TGF‐β. Vimentin expression rises in mesenchymal cells, and lung remodeling and fibrosis are caused by excessive collagen deposition, underscoring the crucial roles these proteins play in the development of disease [44]. Other major proteins involved in the ECM deposition are MMPs. MMP‐2 and MMP‐9, which are mainly produced by Thy‐1 negative lung fibroblasts triggered by TGF‐β1, control fibrocyte migration and ECM remodeling in the course of IPF, encouraging fibroblast accumulation and fibrogenic feedback loops [45].

**FIGURE 5:**
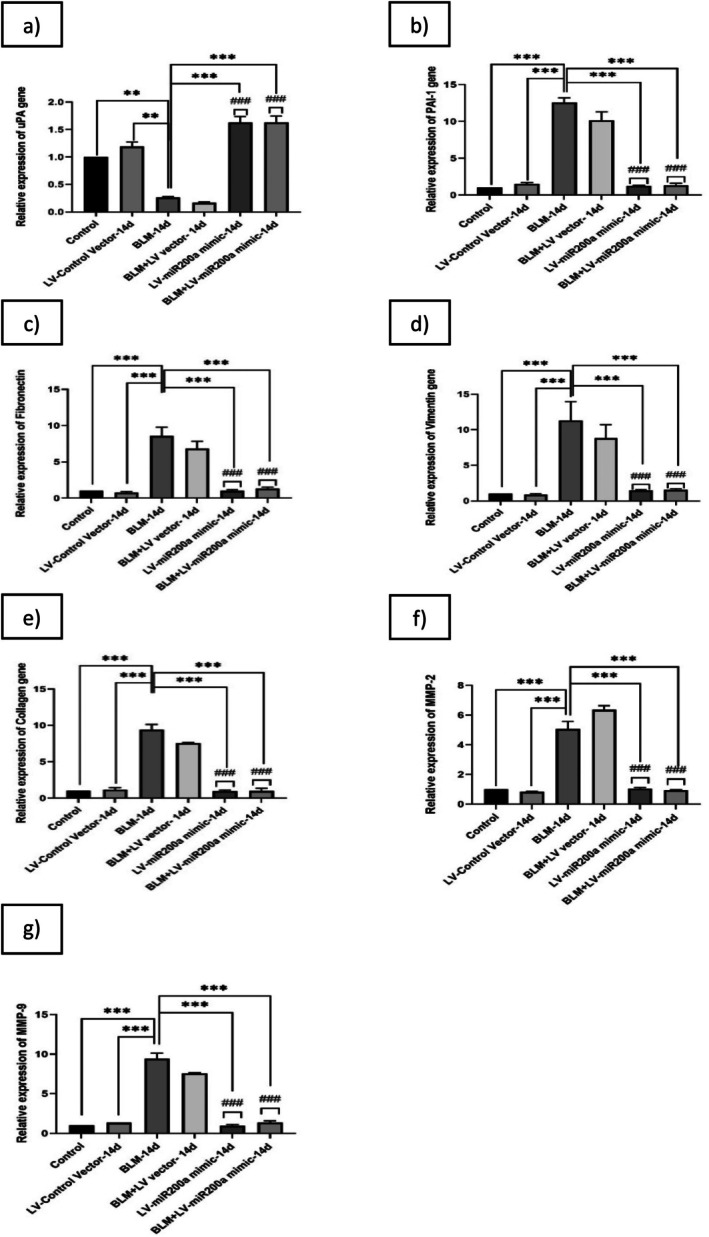
C57BL/6 Mice were exposed to BLM (3 units/kg body wt), and then treated with LV‐miR‐200a mimic after 24 h. Mice were sacrificed after the 14th day of BLM exposure and mice lungs were collected. Total RNA was isolated from the lung homogenate and analyzed by RT‐PCR by using (a) uPA, (b) PAI‐1, (c) Fibronectin, (d) Vimentin, (e) Collagen, (f) MMP‐2, and (g) MMP‐9 gene specific primers. Relative gene expressions were compared with β‐Actin, the relative fold changes are represented graphically. Level of significance: *Indicates significance in comparison with BLM and # indicates significance in comparison with BLM + LV group (*p* < 0.001), (Mean ± SD, *n* = 6).

**FIGURE 6:**
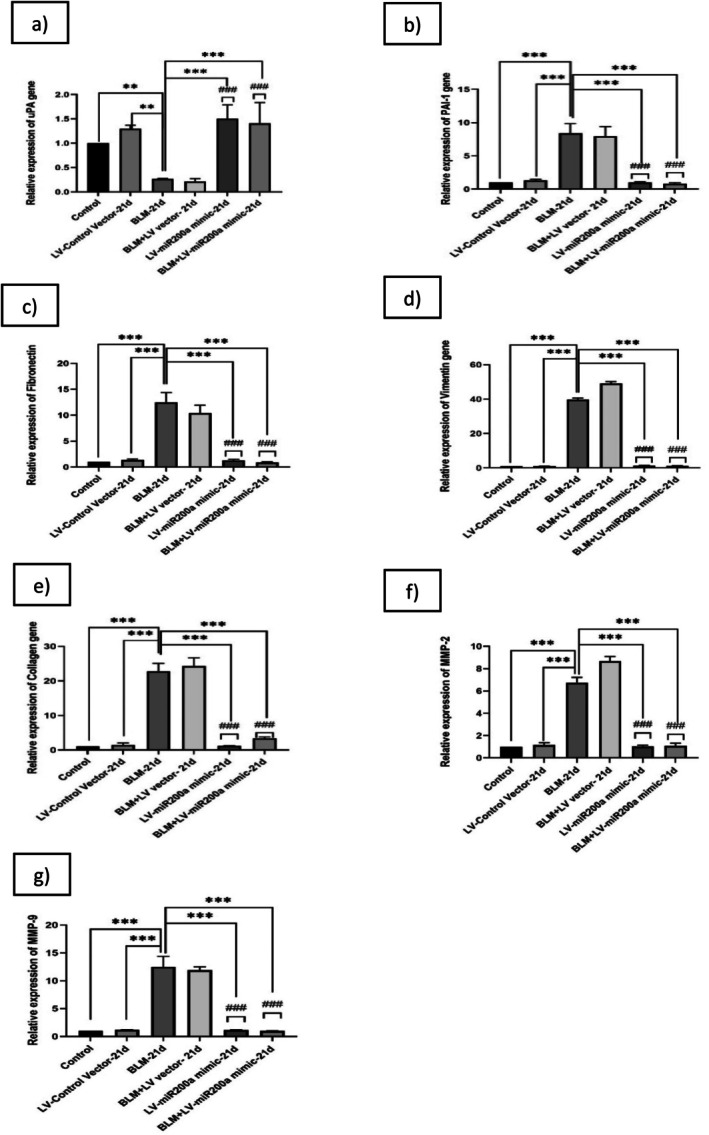
C57BL/6 Mice were exposed to BLM (3 units/kg body wt) and then treated with LV‐miR‐200a mimic after 24 h. Mice were sacrificed after 21st day of BLM exposure, and mice lungs were collected. Total RNA was isolated from the lung homogenate and analyzed by RT‐PCR using (a) uPA, (b) PAI‐1, (c) Fibronectin, (d) Vimentin, (e) Collagen, (f) MMP‐2, and (g) MMP‐9 gene specific primers. Relative gene expressions were compared with β‐Actin; the relative fold changes are represented graphically. Level of significance: *Indicates significance in comparison with BLM and # indicates significance in comparison with BLM + LV group (*p* < 0.001) (Mean ± SD, *n* = 6).

To check the effect of miR‐200a in reversing the dysregulated fibrinolytic system levels and regulating the uncontrolled EMT and ECM deposition in in vivo, the C57BL/6 mice were exposed to BLM at a dose of 3 units/kg body weight and treated with the LV‐miR‐200a mimic 24 h post‐BLM exposure. Mice were sacrificed on the 14th and 21st days post‐BLM exposure, and lung tissues were collected for analysis. Total RNA was extracted from lung homogenates and analyzed using RT‐PCR with gene‐specific primers for uPA, PAI‐1, fibronectin, vimentin, collagen, MMP‐2, and MMP‐9. BLM administration in C57BL/6 mice resulted in significant alterations in genes associated with the fibrinolytic system, ECM deposition, and EMT. uPA expression was markedly reduced (~0.2‐fold, *p* < 0.001; Figures 5a and 6a), while PAI‐1 was increased (~8.4‐fold, *p* < 0.001; Figures 5b and 6b), indicating an imbalance consistent with fibrosis progression. Fibronectin (~8.5‐fold, *p* < 0.001; Figures 5c and 6c), vimentin (~11.3‐fold, *p* < 0.001; Figures 5d and 6d), collagen (~25.8‐fold, *p* < 0.001; Figures 5e and 6e), MMP‐2 (~5.05‐fold, *p* < 0.001; Figures 5f and 6f), and MMP‐9 (~9.4‐fold, *p* < 0.001; Figures 5g and 6g) were all upregulated in the BLM group, reflecting enhanced ECM accumulation and mesenchymal activity.

Treatment with LV‐miR‐200a mimic attenuated these transcript‐level changes. uPA expression increased (~1.6‐fold at day 14; ~1.4‐fold at day 21, *p* < 0.001; Figures 5a and 6a), while PAI‐1 was reduced (~1.3‐fold at day 14; ~0.8‐fold at day 21, *p* < 0.001; Figures 5b and 6b), suggesting a shift towards enhanced fibrinolytic activity. Fibronectin expression decreased from ~8.5‐fold in BLM to ~1.2‐fold at day 14 and ~0.9‐fold at day 21 (*p* < 0.001; Figures 5c and 6c). Vimentin was lowered from ~11.3‐fold in BLM to ~1.5‐fold at day 14 and ~1.1‐fold at day 21 (*p* < 0.001; Figures 5d and 6d). Collagen expression was reduced from ~25.8‐fold in BLM to ~1.1‐fold at day 14 and ~3.4‐fold at day 21 (*p* < 0.001; Figures 5e and 6e). Similarly, MMP‐2 and MMP‐9 were suppressed from ~5.05 and ~9.4‐fold in BLM to ~0.9 and ~1.3‐fold at day 14, and from ~6.7 and ~12.5‐fold in BLM to ~1.1 and ~0.9‐fold at day 21 (*p* < 0.001; Figures 5f,g and 6f,g).

These findings show that miR‐200a overexpression modulates the mRNA expression of genes involved in fibrinolysis, ECM deposition, and EMT in pulmonary fibrosis. While these transcript‐level changes are consistent with reduced fibrotic activity, further studies at the protein level and functional assays are required to confirm restoration of fibrinolytic activity, ECM remodeling, and EMT regulation.

### 4.6 LV‐miR‐200a Mimic Treatment in BLM‐Induced Pulmonary Fibrosis Modulates Fibrinolytic Balance and ECM Remodeling at the Protein Levels

Early in fibrosis, inflammatory cells create more MMP‐2 and MMP‐9, which are subsequently produced by epithelial cells. They promote remodeling and inflammation by breaking down ECM. MMP‐2 promotes later fibrotic development and tissue healing, which propels the advancement of fibrosis, whereas MMP‐9 targets basement membranes early [32]. Myofibroblast formation and the advancement of the disease are facilitated by the partial endothelial‐to‐mesenchymal transition that is induced by the fibrotic ECM in pulmonary fibrosis [46, 47]. MMPs are essential for ECM remodeling and this transition, highlighting their significance in the pathophysiology of IPF and possible targets for treatment [48, 49].

**FIGURE 7:**
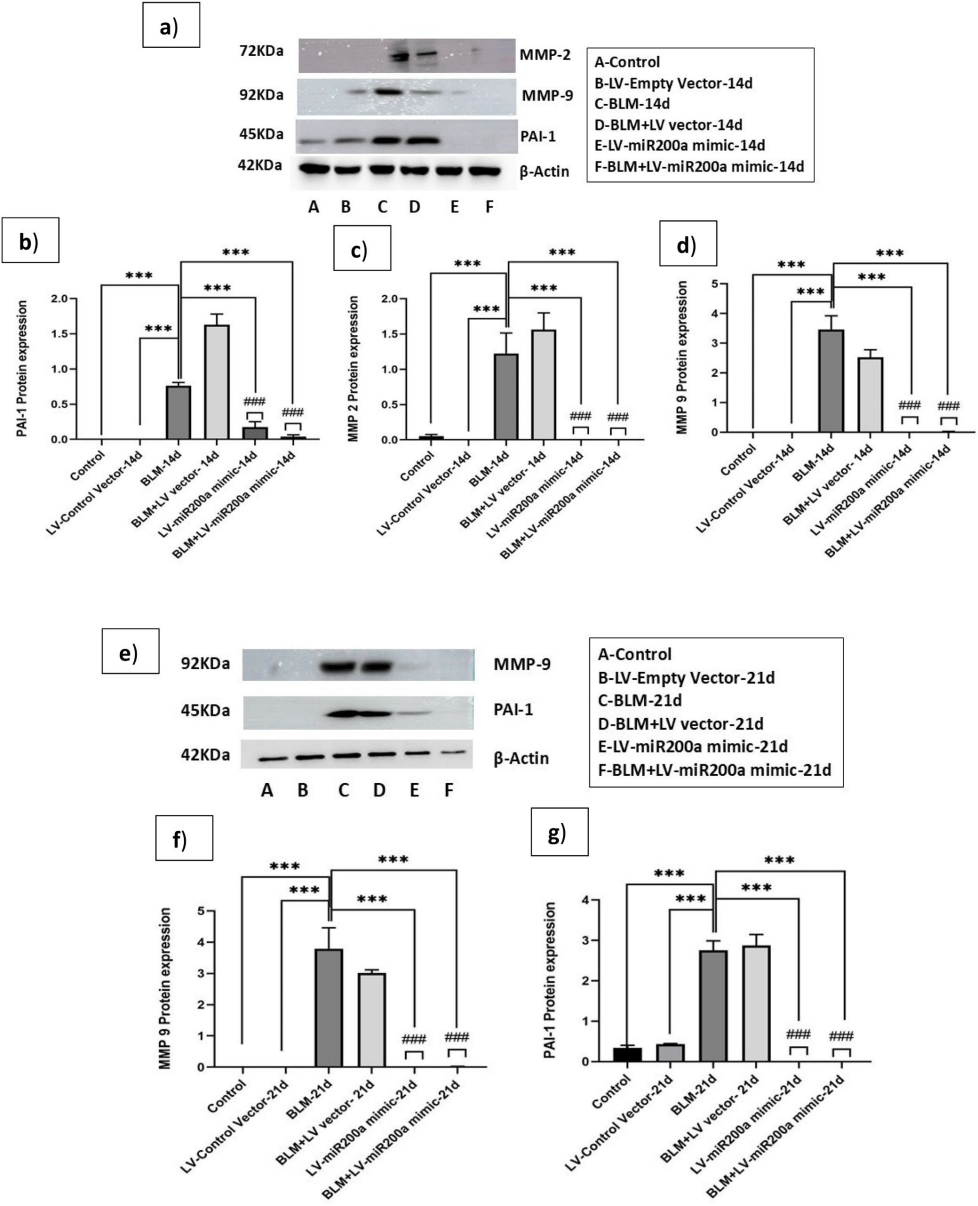
Western blot analysis was performed with protein of C57BL/6 mice sacrificed on the 14th and 21st day of BLM exposure. The 14th day time point (a) Western blot image, (b) PAI‐1, (c) MMP‐9, and (d) MMP‐2; and (e) 21st day time point (f) PAI‐1 and (g) MMP‐9. Blots were normalized using β‐Actin antibody. The densities of individual bands were compiled and normalized against corresponding values of β‐Actin loading controls. *p* < 0.001 will be considered significant. *Indicates significance in comparison with BLM and # indicates significance in comparison with BLM + LV group (*n* = 6).

Consistent with this, overexpression of miR‐200a in the BLM‐induced fibrosis model has been shown to restore fibrinolytic balance and inhibit EMT at the gene level. To validate the transcript‐level findings and assess protein regulation and localization, Western blot and immunofluorescence analyses were performed in BLM‐treated mice with or without LV‐miR‐200a mimic to C57BL/6 mice and analyzed the expression levels of PAI‐1, MMP‐2, and MMP‐9 proteins by western blot for both 14 (Figure 7a) and 21 days (Figure 7e) following BLM treatment. After 14 days of BLM treatment, PAI‐1 expression exhibited a significant ~1.6‐fold increase (*p* < 0.001; Figure 7b). However, miR‐200a overexpression after 24 h of BLM treatment in mice has inhibited PAI‐1 levels, suggesting a shift towards increased matrix degradation and fibrosis resolution. MMP‐2 (Figure 7c) and MMP‐9 (Figure 7d) levels were significantly elevated by ~1.4 and ~3.4‐fold change (*p* < 0.001), respectively, during the fibrosis condition induced by BLM. Conversely, LV‐miR‐200a mimic treatment in the BLM‐induced IPF model reduced the expression of MMP‐2 and MMP‐9, pointing to an active tissue repair and remodeling process that was actively regulated. At the 21‐day mark, PAI‐1 levels (Figure 7f) remained lower in the BLM + LV‐miR‐200a mimic‐treated group compared to the BLM‐induced fibrosis group, showing a ~2.2‐fold difference (*p* < 0.001), consistent with ongoing matrix degradation. Moreover, MMP‐9 expressions (Figure 7g) were further attenuated with a ~3.7‐fold difference (*p* < 0.001) relative to the BLM‐treated condition, indicating continued matrix remodeling and repair processes in a controlled manner.

**FIGURE 8:**
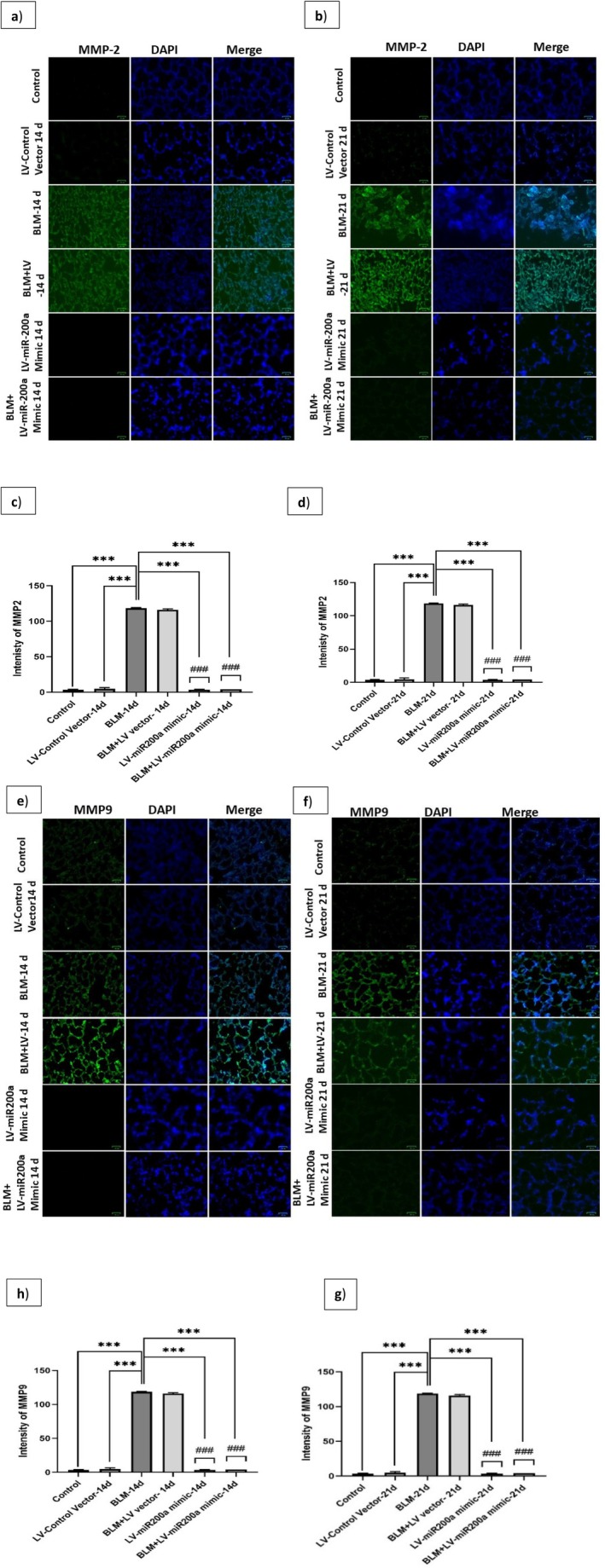
C57BL/6 mice were exposed to BLM (3 units/kg body wt) and then treated with LV‐miR‐200a mimic after 24 h. Mice were sacrificed after the 14th and 21st of BLM exposure, and mice lungs were collected. Mice lung tissue sections (3.5 μm) were subjected to immunofluorescence staining to analyze the levels of MMP‐2 (a) 14th day and (b) 21st day. MMP‐9 (e) 14th day and (f) 21st day. The representative photo‐micrographic images are shown. The intensity bar graph of MMP‐2 (c) 14th day and (d) 21st day, MMP‐9 (g) 14th day and (h) 21st day represents the regulatory effect of LV‐miR200a mimic on MMP‐2 and MMP‐9. Level of significance: *Indicates significance in comparison with BLM and # indicates significance in comparison with BLM + LV group (*p* < 0.001), (Mean ± SD, *n* = 6). Magnification ×400.

To further validate these protein‐level changes and assess their spatial distribution within lung tissue, we performed immunofluorescence analysis. In the BLM‐induced fibrosis model, treatment with LV‐miR‐200a significantly suppressed MMP‐2 (~16.4‐fold reduction at 14 days; ~18.7‐fold reduction at 21 days) and MMP‐9 (~27.6‐fold reduction at 14 days; ~28.3‐fold reduction at 21 days) intensities (Figure 8a–h). To confirm these antifibrotic effects across other markers, we next examined proteins involved in fibrinolysis and ECM deposition. Expression of uPA (~13.7‐fold increase at 14 days; ~14.2‐fold increase at 21 days) and uPAR (~13.9‐fold increase at 14 days; ~15.6‐fold increase at 21 days) was significantly restored compared to the BLM‐only group (Figure S4a–h). In contrast, PAI‐1 (~12.4‐fold reduction at 14 days; ~11.8‐fold reduction at 21 days), TNF‐α (~29.5‐fold reduction at 14 days; ~14‐fold reduction at 21 days), and fibronectin (~12.6‐fold reduction at 14 days; ~18.9‐fold reduction at 21 days) were markedly decreased (Figures S4i–l and S5a–h). EMT regulators ZEB1 (~12.3‐fold reduction at 14 days; ~26.9‐fold reduction at 21 days) and ZEB2 (~13.8‐fold reduction at 14 days; ~27.4‐fold reduction at 21 days) followed the same trend (Figure S6a–h). These findings confirm that miR‐200a suppresses ECM remodeling through MMP regulation while simultaneously restoring fibrinolytic activity and limiting EMT progression in fibrotic tissue.

## 5 Discussion

miRNAs play a crucial role in regulating processes involved in pulmonary diseases such as IPF and ALI [50, 51, 52]. Among them, miR‐200a is particularly significant in IPF due to its ability to inhibit the expression of MMPs. This inhibition helps limit ECM degradation and contributes to reversing the fibrotic changes observed in IPF [53, 54]. In ALI, miR‐200a, along with miR‐200b, has been shown to restore the balance of the fibrinolytic system, which is essential for controlling the inflammatory response and promoting tissue repair. Building on these findings, our study demonstrates that lentiviral delivery of miR‐200a mimics in BLM‐induced fibrosis effectively regulates MMPs and fibrinolytic components, highlighting its therapeutic potential in PF.

BLM is an oncolytic drug used to treat certain types of cancers, which functions by inducing DNA strand breaks followed by cell death [55]. Despite its effectiveness, BLM can cause serious pulmonary side effects, such as fibrosis and pneumonitis, especially in elderly individuals or those taking large dosages over time. The drug induces fibrosis through ROS generation, oxidative stress, and tissue damage. This leads to epithelial cell injury, an inflammatory response, and EMT, transforming damaged cells into mesenchymal cells, causing excessive ECM components [56]. In our model, intranasal BLM administration produced acute inflammation at 3–7 days and fibrosis at 14–21 days [57, 58]. This timeline allowed us to investigate the role of miR‐200a in regulating MMPs and fibrinolytic markers during both the inflammatory and fibrotic stages. Lentiviral delivery ensured stable and sustained miR‐200a expression, enabling consistent modulation of fibrotic markers throughout the study period [59, 60, 61, 62, 63].

The morphological changes in fibrosis condition mark the severity of the disease [64]. Hence, we decided to perform H and E staining to understand the level of substantial derangement that happened in the lung architecture after BLM ingestion. H&E staining revealed infiltration of inflammatory markers and alveolar thickening in BLM‐treated lungs at 14 and 21 days. In contrast, LV‐miR‐200a treatment reversed these changes, restoring lung morphology towards control levels. This observation supports previous evidence that miR‐200 family members reconstruct tissue morphology in renal fibrosis [65], and regulate cell proliferation and organ development [66, 67]. The grading of fibrosis in the BLM + LV‐miR‐200a group was markedly reduced compared to BLM alone, further confirming the curative role of miR‐200a in PF.

In correlation with the disturbed lung morphology the term fibrosis collides with the collagen deposition since collagen cross‐linking is contributing to the development of fibrosis [68, 69]. Collagen and other ECM markers will significantly rise in their expression and uncontrolled activation results in the lung tissue remodeling [70, 71, 72]. MT staining demonstrates the substantial amount in deposition of collagen in the lung tissue sectioning. His reduction highlights the therapeutic potential of miR‐200a in negatively regulating collagen deposition, consistent with its broader role in ECM remodeling [73, 74].

Studies have proven the importance of the fibrinolytic system in the progression of PF. In our previous study, we have quoted the involvement of uPA, uPAR, and PAI‐1 during ALI, and the interconnection between miR‐200a and miR‐200b in the regulation of the fibrinolytic system. Here in this study, we ascertained the pivotal role of miR‐200a in the regulation of the fibrinolytic system during PF. LV‐miR‐200a upregulated the uPA and uPAR levels while downregulating the levels of PAI‐1, reversing the PF. A crucial ECM protein called fibronectin encourages fibroblasts to become myofibroblasts, and the excessive deposition of ECM, including fibronectin, by these myofibroblasts is the cause of lung tissue stiffening and scarring [75, 76], was elevated in BLM‐treated lungs but suppressed by miR‐200a. Vimentin, another EMT marker, showed a similar pattern: elevated in BLM‐treated mice but reduced after LV‐miR‐200a intervention. Collagen expression also decreased in the treated group. These observations underscore the comprehensive impact of miR‐200a in regulating fibrotic markers, suggesting its potential to counteract ECM build‐up and fibrosis‐related changes. Although transcriptional changes in fibronectin and vimentin were observed, protein‐level validation was not performed in this study. As transcriptional output does not always correspond with protein abundance, future work will include protein‐level analysis to fully establish the translational relevance of these findings.

MMPs, including MMP‐2 and MMP‐9, are critical enzymes involved in ECM remodeling [77]. They play a dual role in PF, contributing to both the progression and resolution of fibrotic processes [78]. During fibrosis, MMPs are often dysregulated, leading to excessive ECM degradation or accumulation, which disrupts normal lung architecture and function [79, 80, 81]. Our study showed that LV‐miR‐200a reduced PAI‐1, MMP‐2, and MMP‐9 expression at both 14 and 21 days, supporting controlled tissue repair and matrix degradation. By day 21, sustained suppression of PAI‐1 and MMP‐9 reinforced ongoing repair processes, confirming the therapeutic role of miR‐200a in regulating ECM dynamics.

Immunofluorescence studies have been widely utilized in PF research to visualize protein expression and cellular localization. For instance, a study highlighted the role of NR2F2 in alleviating epithelial cell senescence and fibroblast activation in BLM‐induced fibrosis. Similarly, research on the NRF2 pathway demonstrated its antioxidative and anti‐inflammatory effects in silica‐induced pulmonary fibrosis, using immunofluorescence to confirm reduced oxidative stress and fibroblast activation [82]. Another investigation explored the interaction between ficolin‐1 and TGF‐β1, employing immunofluorescence to demonstrate its direct binding and therapeutic efficacy in mitigating fibrosis [83].

In the context of miR‐200a, previous studies have shown its ability to regulate TGF‐β signaling and EMT processes, which are central to PF progression. For example, miR‐200a was found to inversely correlate with Hedgehog and TGF‐β pathways, orchestrating antifibrotic effects in BLM‐induced fibrosis [84]. These findings are consistent with the observed reduction in fibrotic markers in the current study, further supporting the therapeutic potential of LV‐miR‐200a mimics. Although our study demonstrates consistent transcriptional and protein‐level changes in EMT and ECM markers following miR‐200a overexpression, these findings are correlative rather than causal. Direct mechanistic validation, such as knockdown of pathway members in cell‐based models, will be required to establish causality and fully justify the role of miR‐200a in EMT and ECM modulation. We acknowledge this limitation and highlight it as an important future direction.

Overall, the immunofluorescence results provide compelling evidence of the spatial and functional modulation of fibrotic markers by miR‐200a mimics. Combined with insights from similar studies, these findings underscore the promise of miR‐200a as a targeted therapeutic approach for pulmonary fibrosis, offering a novel avenue for ECM regulation and tissue repair.

## 6 Conclusion

In the present study, we examine the influence of miR‐200a on the fibrinolysis and MMPs. Through a combination of gene expression analysis, protein detection, histochemistry staining and immunofluorescence analysis, we have provided abundant evidence that lentivirus represented miR‐200a mimics restore fibrinolytic equilibrium, repress ECM degradation, and diminish EMT markers in BLM‐induced pulmonary fibrosis.

The overexpression of miR‐200a positively regulates the fibrinolytic system by upregulating uPA and uPAR while downregulating PAI‐1, thereby reversing the profibrotic environment. In parallel, miR‐200a reduces fibronectin, collagen, vimentin, MMP‐2, and MMP‐9, confirming its broad role in limiting ECM deposition and tissue stiffening. These findings extend previous reports of miR‐200 family members in renal fibrosis and ALI, demonstrating their relevance in pulmonary fibrosis as well.

Importantly, the lentiviral delivery system ensured stable and sustained expression of miR‐200a, allowing us to capture its therapeutic effects across both the inflammatory and fibrotic stages. This highlights the translational potential of miR‐200a mimics as a long‐term antifibrotic strategy. While our study is limited to a murine model, the consistent modulation of multiple fibrotic pathways underscores the promise of miR‐200a as a targeted therapeutic approach.

Future studies should explore alternative delivery systems, long‐term efficacy, and validation in human IPF tissues. Collectively, our findings position miR‐200a as an innovative therapeutic candidate for pulmonary fibrosis, offering a novel avenue for ECM regulation, fibrinolytic balance, and tissue repair.

## Author Contributions

T.M. Jeena have performed conceptualization, experimental design, in vitro and in vivo experiments, molecular analysis, data analysis, figure preparation, manuscript preparation and revision. C. Rakshitha helped with in vivo experiment contribution and critical review of the manuscript. Dr. Akarsha B. Jain assisted with animal experiment assistance and experimental execution contribution. Vaishnavi and Fathima Razana helped in in vivo experiment assistance and sample processing. Aleena Varughese supported in vivo work and animal handling. Dr. M. Fathimath Muneesa provided manuscript editing and review. Yashodhar P. Bhandary served as project sponsor, funding, and final manuscript approval. Each author has read and approved the final version of the manuscript.

## Funding

Yenepoya University Seed Grant (YU/Seed grant/131‐2022) has supported the entire study financially.

## Conflicts of Interest

The authors declare no conflicts of interest.

## Supporting information


**Appendix S1:** fba270096‐sup‐0001‐AppendixS1.docx.

## Data Availability

The data information generated through and/or analyzed through the present study are available from the corresponding author upon reasonable request. No custom code was employed in the current study.
