# Supplementary material for: Therapeutic Potential of Lentiviral miR‐200a Mimics in Regulating Fibrinolysis and EMT Markers During Pulmonary Fibrosis
Source: FASEB Bioadv. 2026 Apr 17;8(4):e70096. doi: 10.1096/fba.2025-00276 (PMC13088896; doi:10.1096/fba.2025-00276)
Supplement: Supplementary file 1 — Appendix S1: fba270096‐sup‐0001‐AppendixS1.docx. [file FBA2-8-e70096-s001.docx]

**Supplementary Documents**

| **Target** | **Source** | **Catalog Number** | **Antibody Type** | **RRID** |
| --- | --- | --- | --- | --- |
| β-actin | Sigma-Aldrich, Japan | A2228 | Polyclonal | RRID:AB_476744 |
| IL-17A | Cell Signaling Technology, USA | 512304 | Polyclonal | RRID:AB_10695774 |
| PAI-1 | Bio-Rad, USA | MAB1796 | Polyclonal | RRID:AB_2128657 |
| uPA | R&D Systems, USA | AF1287 | Polyclonal | RRID:AB_354276 |
| uPAR | R&D Systems, USA | AF807 | Polyclonal | RRID:AB_354401 |
| TGF-β1 | Cell Signaling Technology, USA | 3711 | Polyclonal | RRID:AB_2200250 |
| MMP-2 | R&D Systems, USA | AF902 | Polyclonal | RRID:AB_355398 |
| MMP-9 | R&D Systems, USA | AF909 | Polyclonal | RRID:AB_355402 |
| ZEB1 | R&D Systems, USA | AF5480 | Polyclonal | RRID:AB_10572244 |
| ZEB2 | R&D Systems, USA | AF3486 | Polyclonal | RRID:AB_10572243 |
| Fibronectin | Cell Signaling Technology, USA | 26836 | Polyclonal | RRID:AB_2799026 |
| Vimentin | Cell Signaling Technology, USA | 5741 | Polyclonal | RRID:AB_10695459 |
| Collagen I | R&D Systems, USA | AF6220 | Polyclonal | RRID:AB_1603299 |

**Table S1:** Antibodies Used in This Study with RRID Information

| **Vector Element** | **Utility** |
| --- | --- |
| 5' LTR | 5' Long Terminal Repeat is necessary for lentiviral particle production and integration of the construct into the host cell genome |
| Ψ | Psi packaging sequence allows lentiviral genome packaging using lentiviral packaging systems |
| RRE | Rev Response Element enhances titer by increasing packaging efficiency of full-length lentiviral genomes |
| TRE3G | Inducible promoter with Tetracycline Response Elements which is activated by the Tet-On 3G protein in the presence of doxycycline |
| tGFP or tRFP | TurboGFP or TurboRFP reporter for visual tracking of transduction and expression upon doxycycline induction |
| SMARTvector universal scaffold | Optimized proprietary scaffold based on native primary microRNA in which gene-targeting sequence is embedded |
| Puro^R^ | Puromycin resistance gene permits antibiotic selection of transduced cells |
| 2A | Self-cleaving peptide that enables the expression of both PuroR and Tet-On 3G transactivator from a single RNA pol II promoter |
| Tet-On 3G | Encodes the doxycycline-regulated transactivator protein, which binds to TRE3G only in the presence of doxycycline |
| WPRE | Woodchuck Hepatitis Post-transcriptional Regulatory Element enhances transgene expression in target cells |
| 3' SIN LTR | 3' Self-inactivating Long Terminal Repeat for generation of replication-incompetent lentiviral particles |

**Table S2:** Lentiviral vector elements and utility


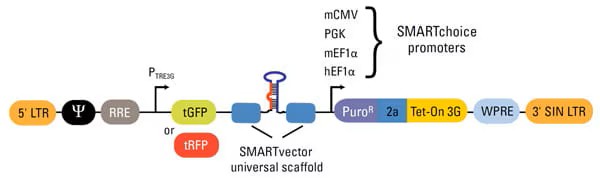


**Figure S1:** Lentiviral Vector image**
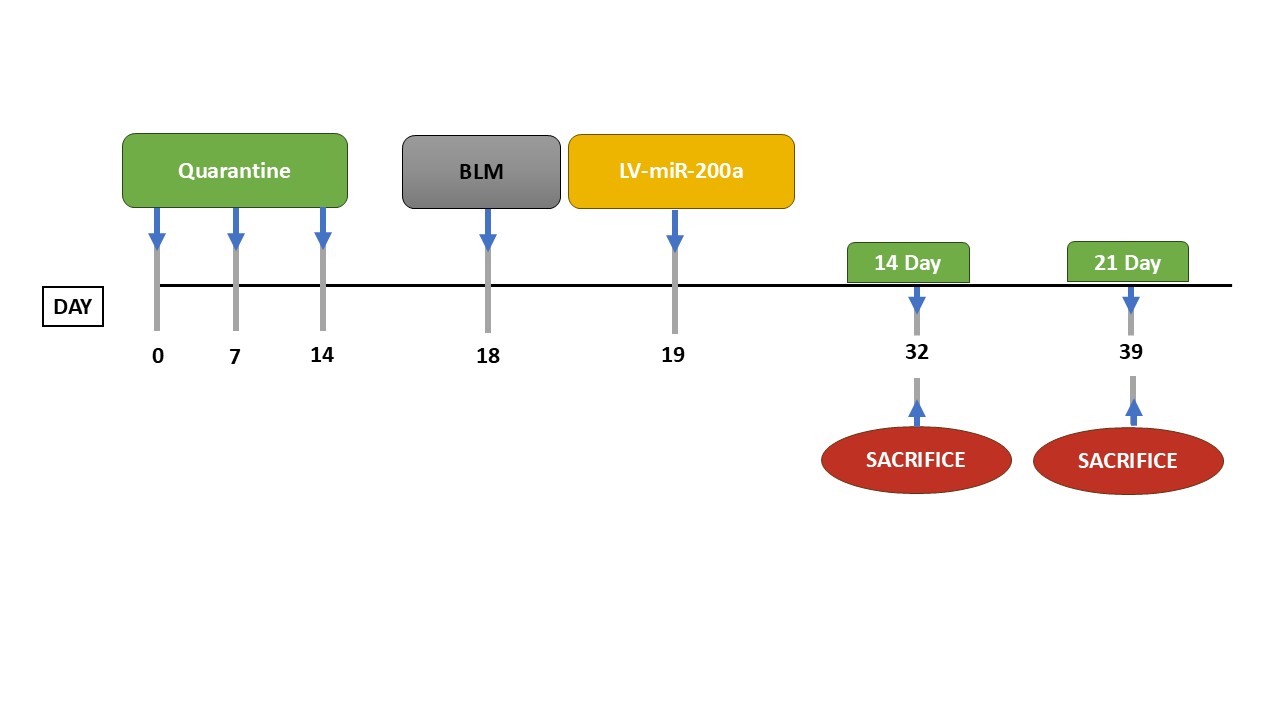
**

**Figure S2:** *In vivo* treatment of BLM, LV-miR-200a flow chart

**
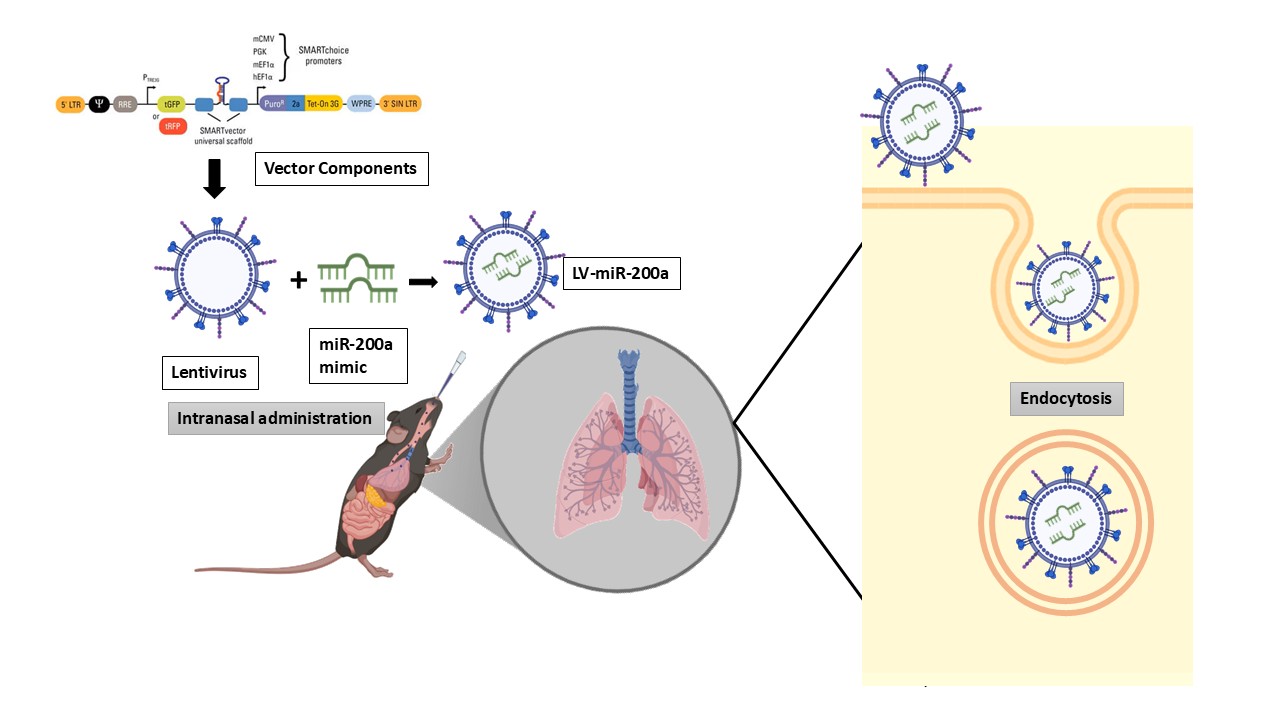
**

**Figure S3:** Overview of intranasal administration of LV-miR-200a

**
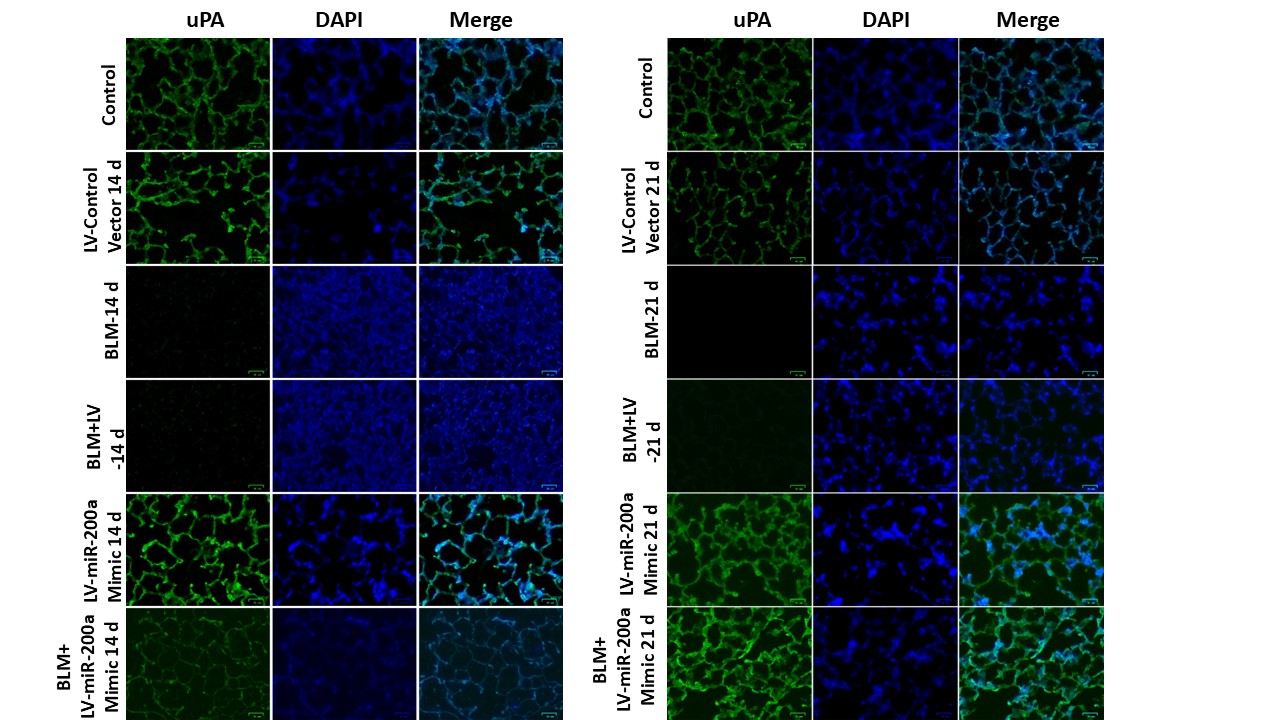
**

**b**)

**a**)

**c**)

**d**)

**
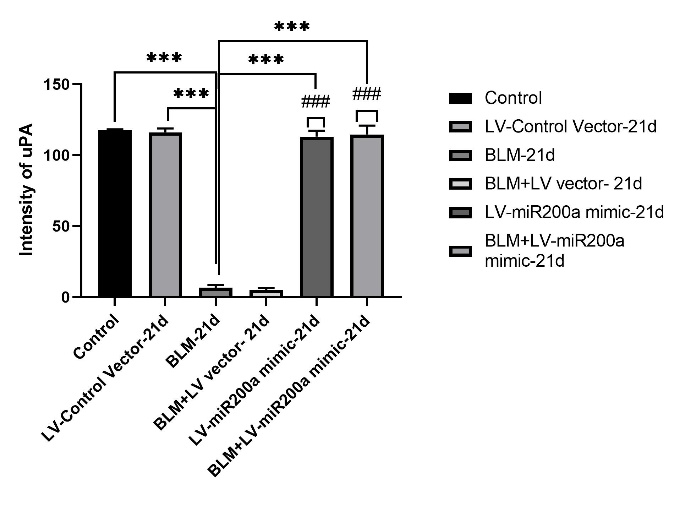

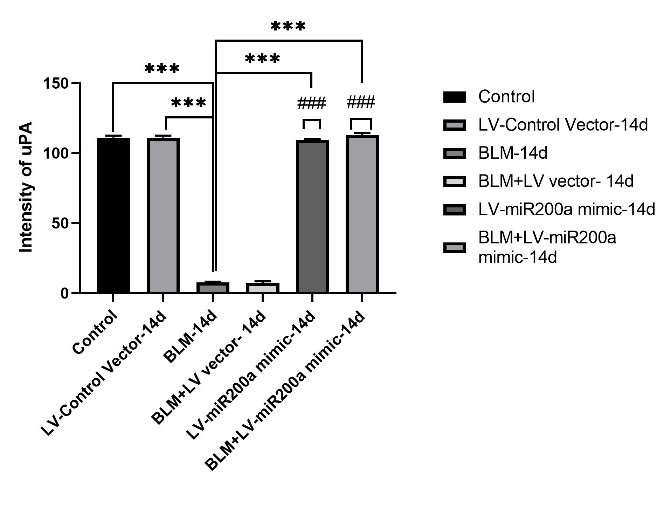
**

**
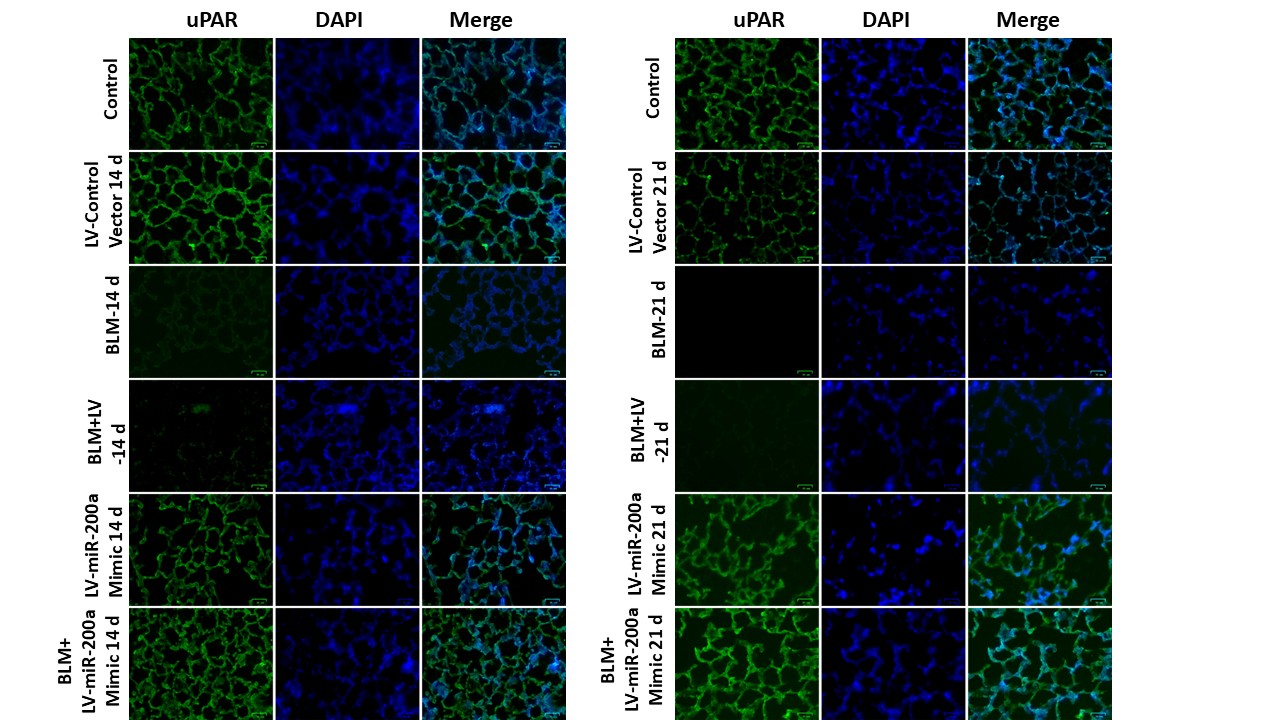
**

**e**)

**f**)

**g**)

**h**)

**
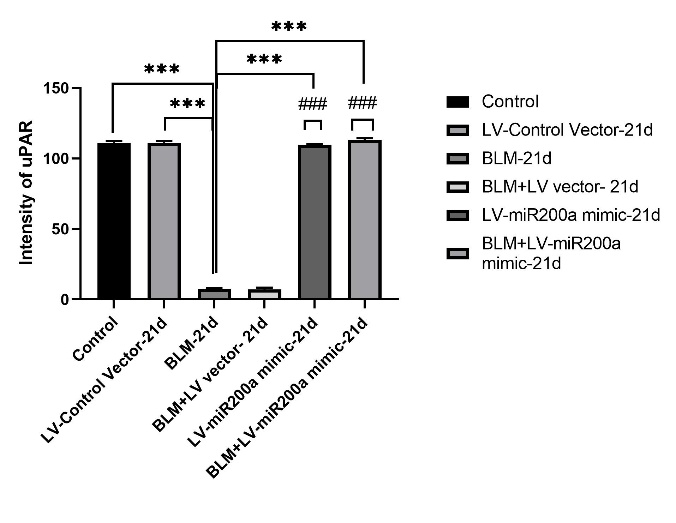

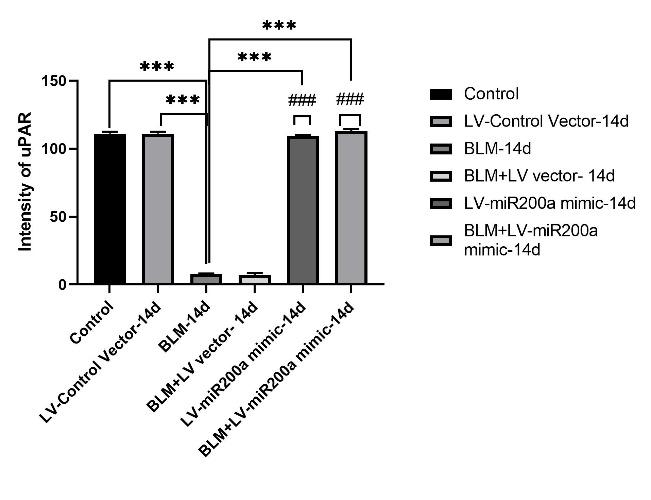
**

**
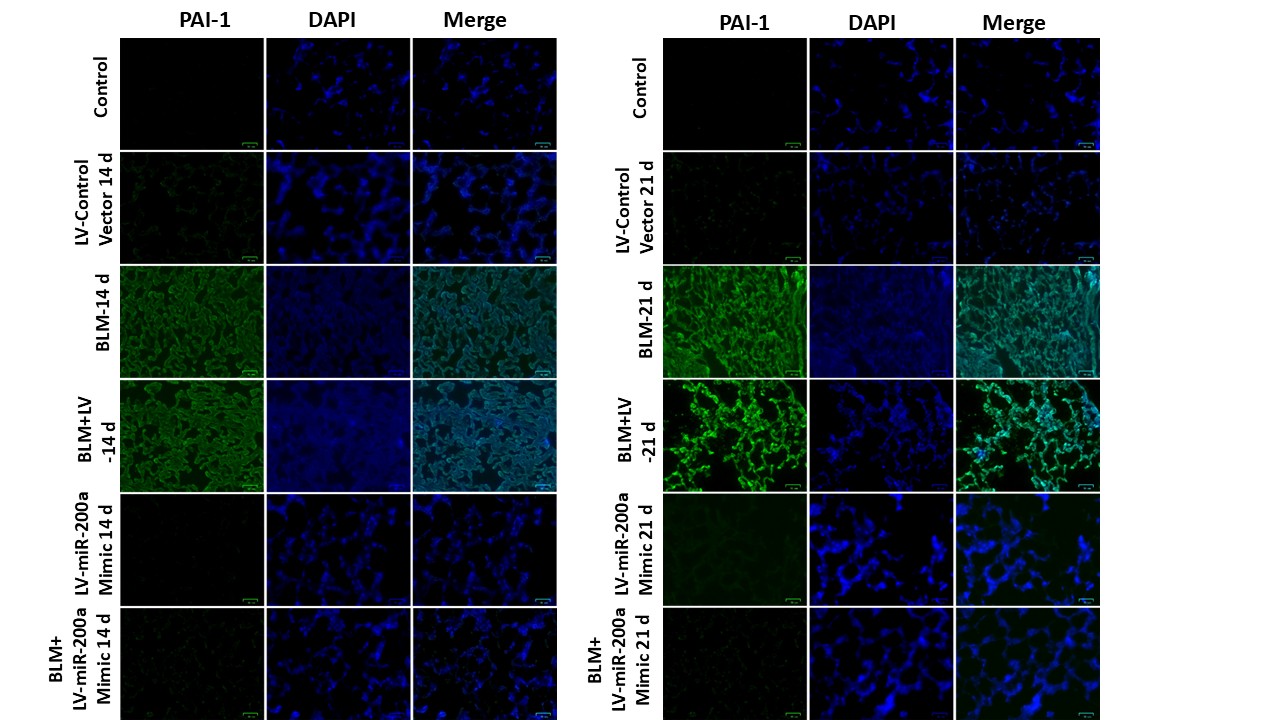
**

**j**)

**i**)

**l**)

**
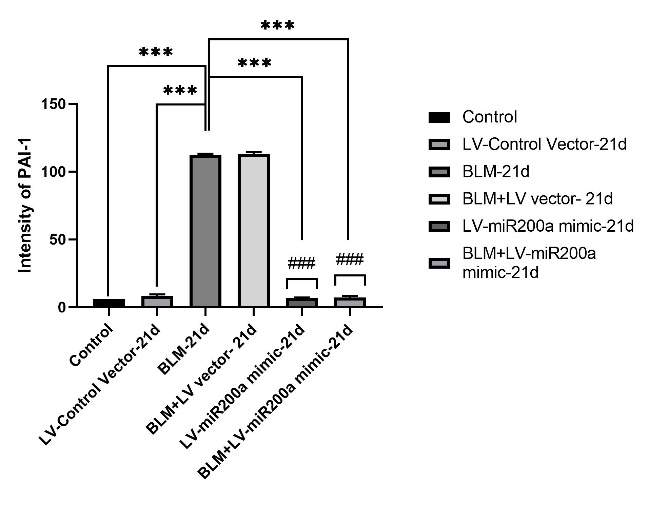

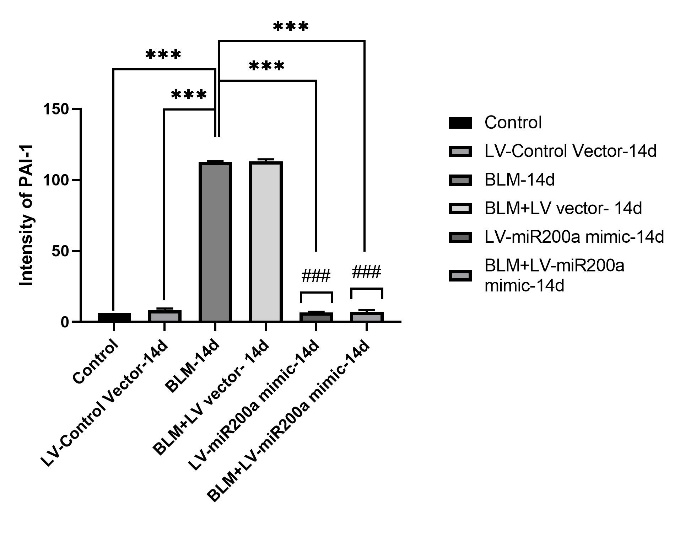
**

**k**)

**Figure S4:** C57BL/6 mice were exposed to BLM (3units/kg body wt), and then treated with LV-miR-200a mimic after 24 h. Mice were sacrificed after 14^th^ and 21^st^ of BLM exposure and mice lungs were collected. Mice lung tissue sections (3.5 μm) were subjected to immunofluorescence staining to analyse the levels of uPA **(a)** 14^th^ day and **(b)** 21^st^ day, uPAR **(e)** 14^th^ day and **(f)** 21^st^ day, PAI-1 **(i)** 14^th^ day and **(j)** 21^st^ day. The representative photo-micrographic images are shown. The intensity bar graph of uPA **(c)** 14^th^ day and **(d)** 21^st^ day, uPAR **(g)** 14^th^ day and **(h)** 21^st^ day, PAI-1 **(k)** 14^th^ day and **(l)** 21^st^ day. represents the regulatory effect of LV-miR200a mimic on uPA, uPAR and PAI-1. Level of significance: *Indicates significance in comparison with BLM and # indicates significance in comparison with BLM+LV group (p<0.001), (Mean ± SD, n = 3) Magnification ×400.

**
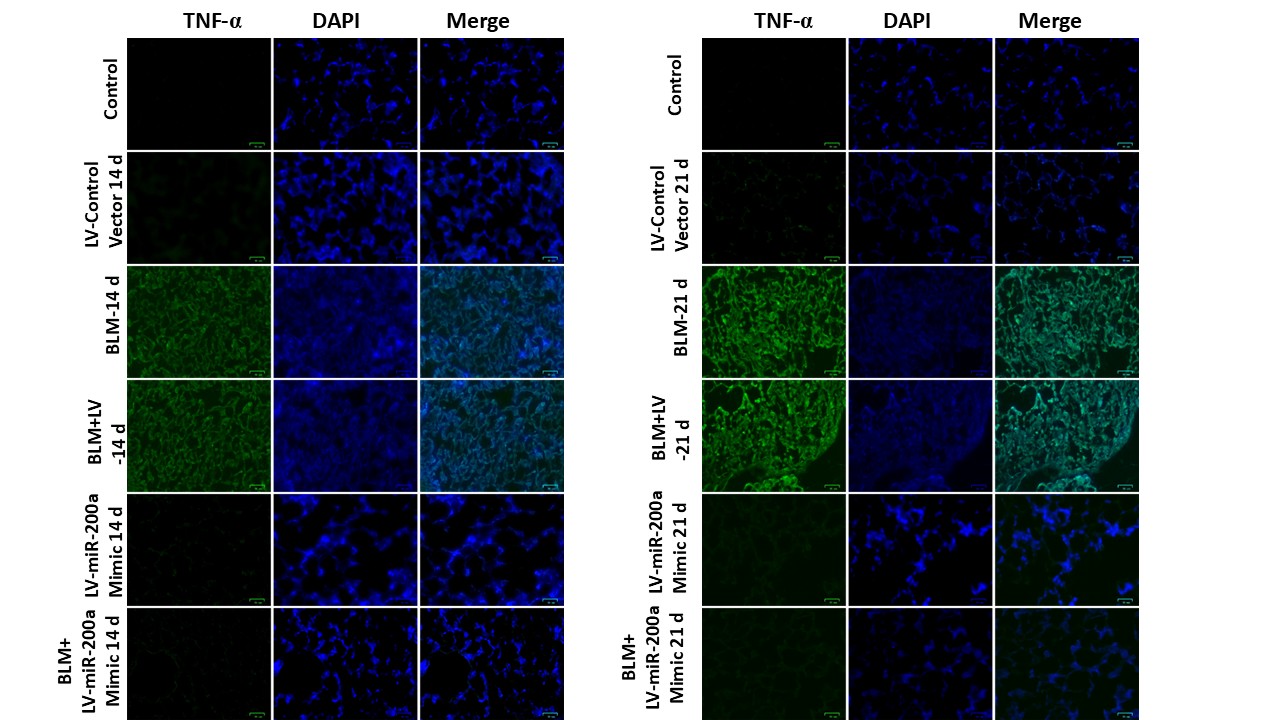
**

**b**)

**a**)

**d**)

**
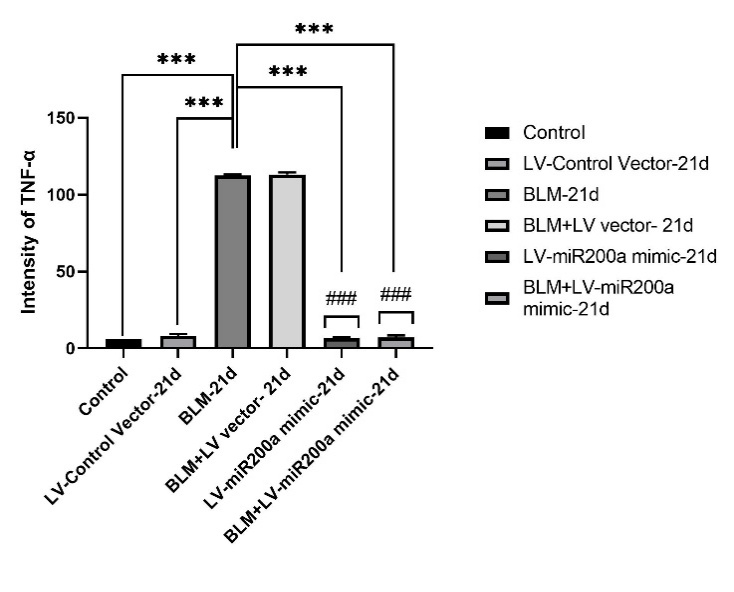

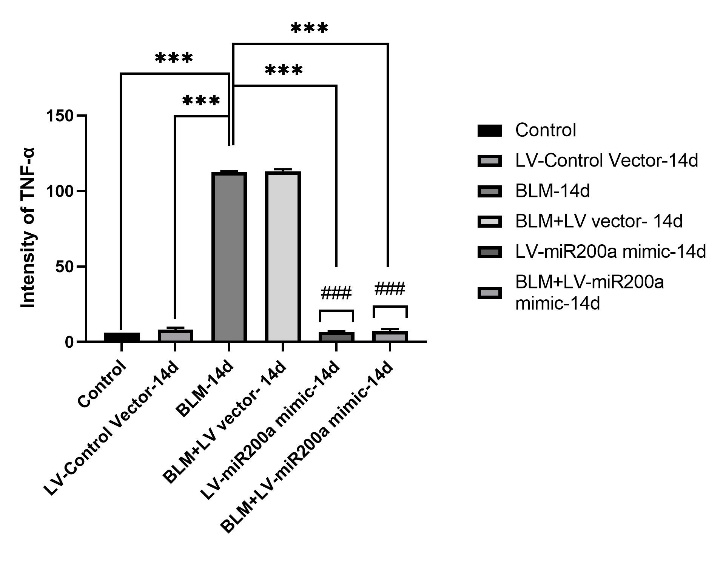
**

**c**)

**
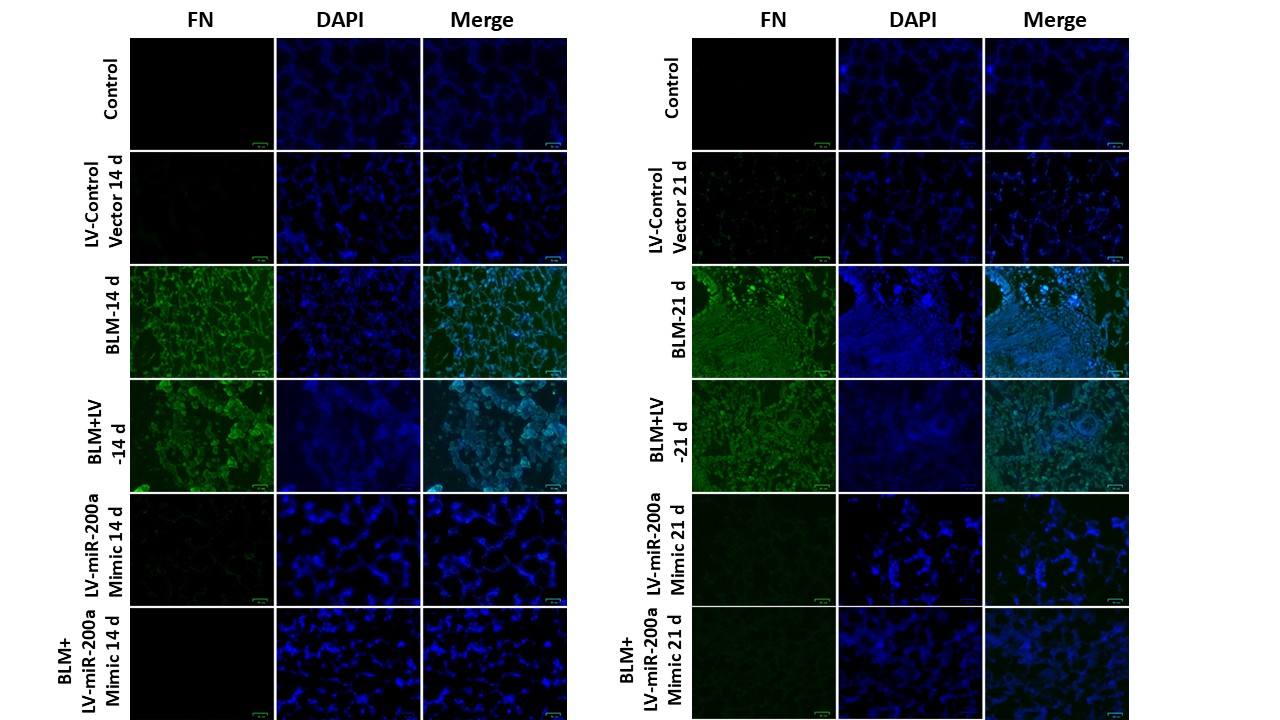
**

**f**)

**e**)

**
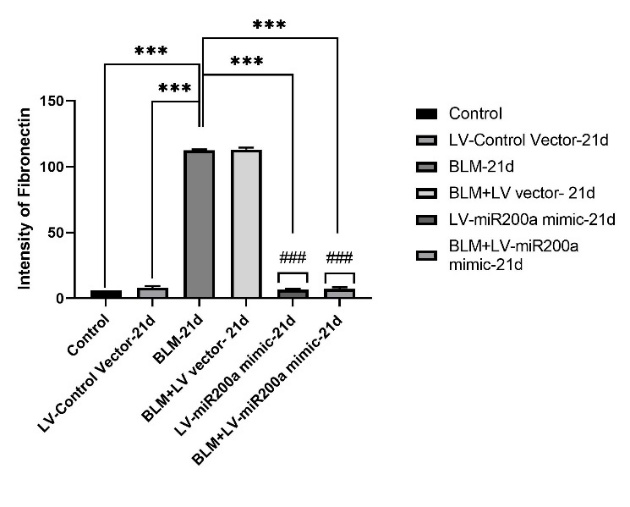

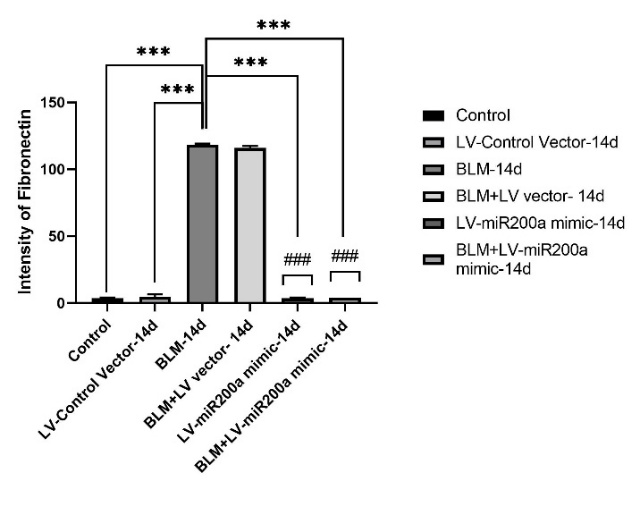
**

**g**)

**h**)

**Figure S5:** C57BL/6 mice were exposed to BLM (3units/kg body wt), and then treated with LV-miR-200a mimic after 24 h. Mice were sacrificed after 14^th^ and 21^st^ of BLM exposure and mice lungs were collected. Mice lung tissue sections (3.5 μm) were subjected to immunofluorescence staining to analyse the levels of TNF-α **(a)** 14^th^ day and **(b)** 21^st^ day. Fibronectin **(e)** 14^th^ day and **(f)** 21^st^ day. The representative photo-micrographic images are shown. The intensity bar graph of TNF-α **(c)** 14^th^ day and **(d)** 21^st^ day, Fibronectin **(g)** 14^th^ day and **(h)** 21^st^ day represents the regulatory effect of LV-miR200a mimic on TNF-α and Fibronectin. Level of significance: *Indicates significance in comparison with BLM and # indicates significance in comparison with BLM+LV group (p<0.001), (Mean ± SD, n = 3). Magnification ×400.

**
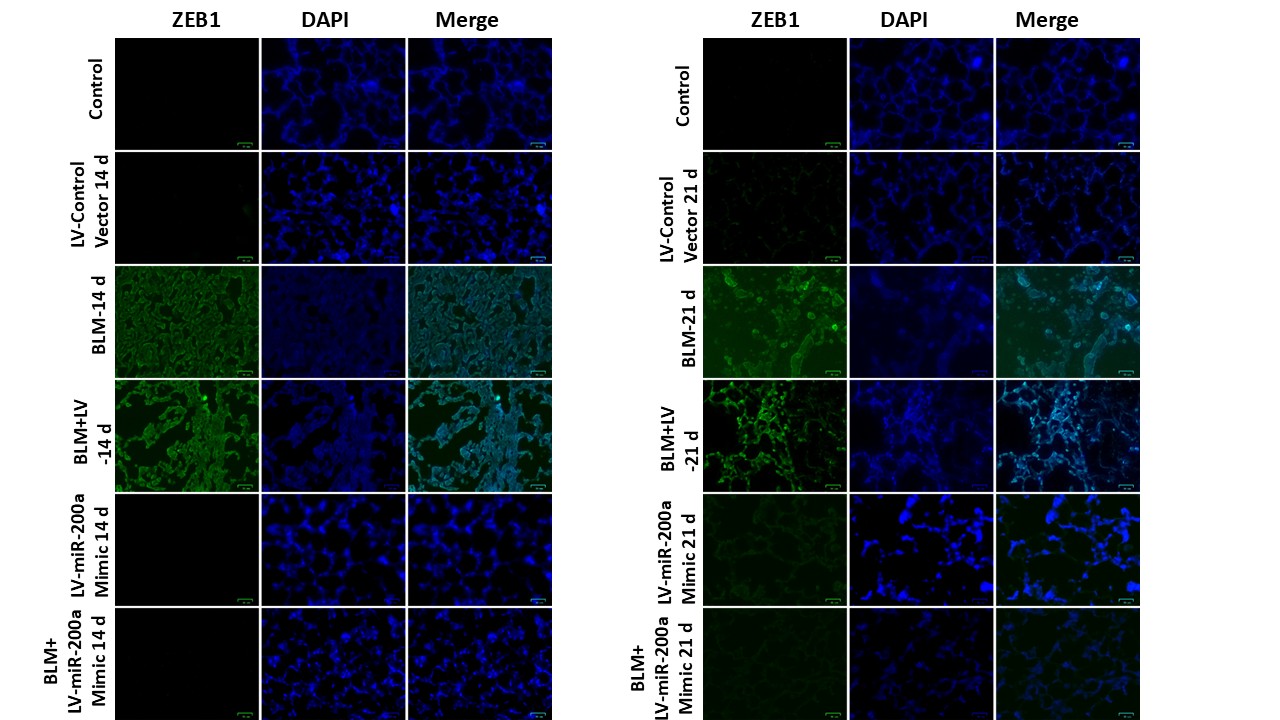
**

**c**)

**d**)

**C**)

**b**)

**a**)

**
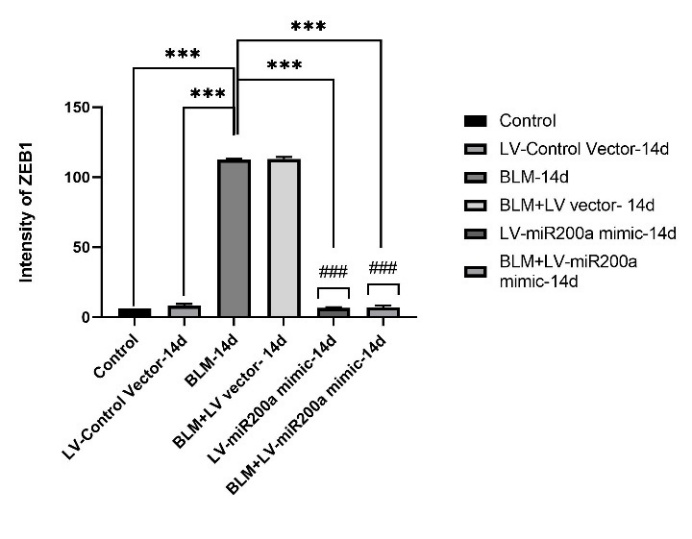

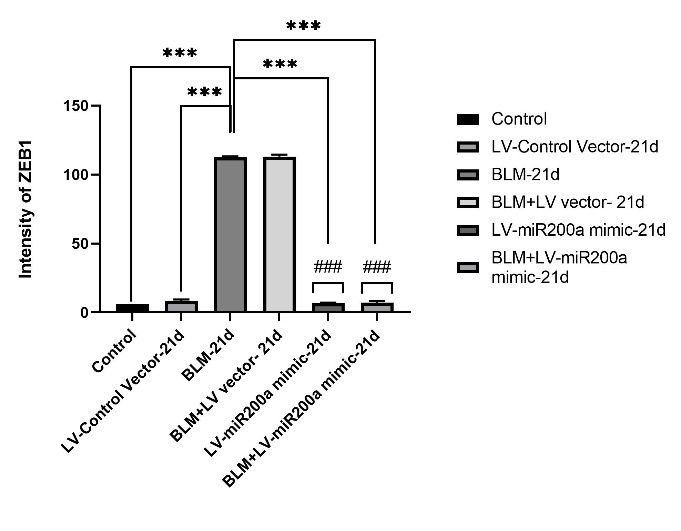
**

**
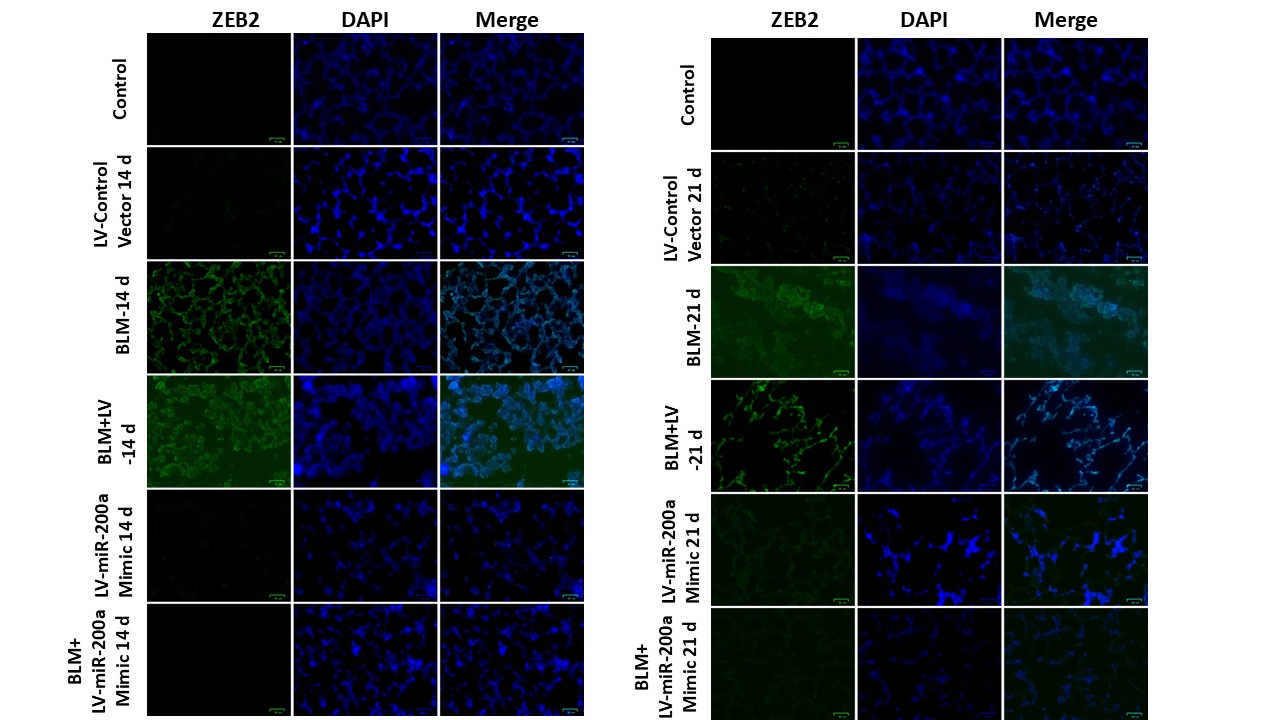
**

**e**)

**f**)

**
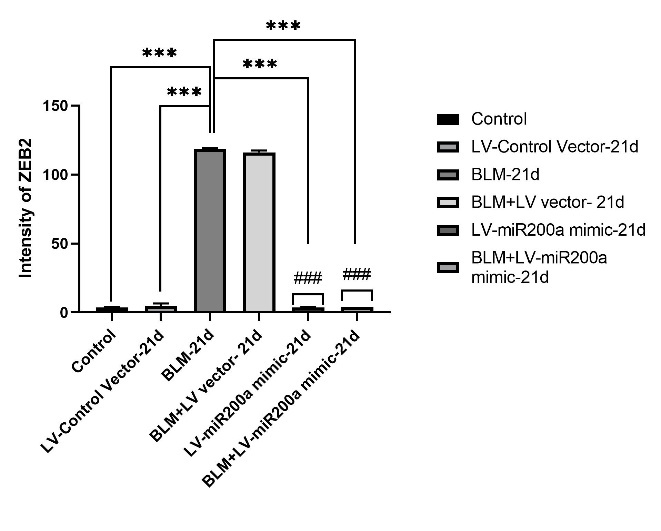

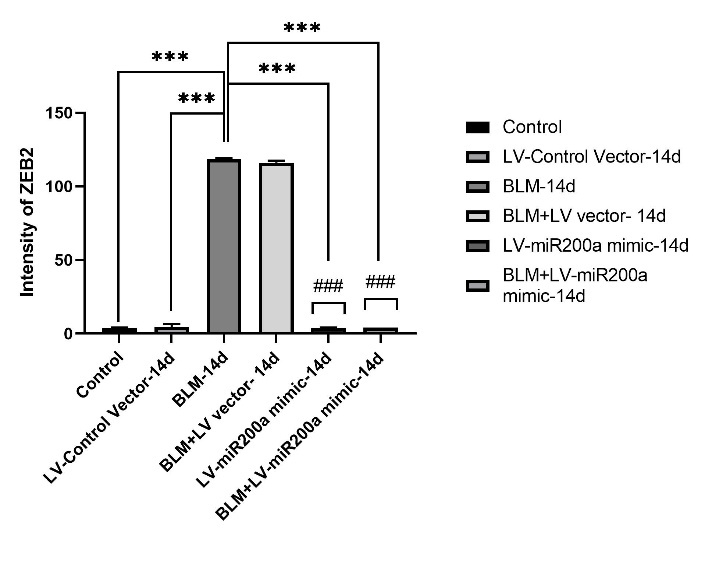
**

**h**)

**g**)

**Figure S6:** C57BL/6 mice were exposed to BLM (3units/kg body wt), and then treated with LV-miR-200a mimic after 24 h. Mice were sacrificed after 14^th^ and 21^st^ of BLM exposure and mice lungs were collected. Mice lung tissue sections (3.5 μm) were subjected to immunofluorescence staining to analyse the levels of ZEB1 **(a)** 14^th^ day and **(b)** 21^st^ day. ZEB2 **(e)** 14^th^ day and **(f)** 21^st^ day. The representative photo-micrographic images are shown. The intensity bar graph of ZEB1 **(c)** 14^th^ day and **(d)** 21^st^ day, ZEB2 **(g)** 14^th^ day and **(h)** 21^st^ day represents the regulatory effect of LV-miR200a mimic on ZEB1 and ZEB2. Level of significance: *p<0.001 compared to BLM exposure (Mean ± SD, n=3). Magnification ×400.
